# Women's Experiences of Breastfeeding Beyond One Year: A Qualitative Systematic Review

**DOI:** 10.1111/mcn.70242

**Published:** 2026-09-01

**Authors:** Thomas Cronin, Noor Ainah Gulzar Ahmad, Noel McCarthy

**Affiliations:** ^1^ School of Public Health, University College Cork, Cork, Ireland, Discipline of Public Health and Primary Care School of Medicine, Trinity College Dublin Dublin Ireland; ^2^ School of Medicine, Trinity College Dublin Dublin Ireland; ^3^ Discipline of Public Health and Primary Care, School of Medicine Trinity College Dublin Dublin Ireland

**Keywords:** breastfeeding, experiences, qualitative, systematic review

## Abstract

Breastfeeding beyond 1 year is recommended by the World Health Organisation and is linked to multiple maternal, infant, and environmental benefits. However, rates frequently remain low in high‐income countries, which may relate to social stigma, work commitments and insufficient support. This review aimed to explore the experiences of women who breastfeed beyond 1 year. Accordingly, a qualitative systematic review following the Joanna Briggs Institute meta‐aggregation approach and reported to the ENTREQ statement was undertaken. Searches were conducted in Ovid MEDLINE, Ovid EMBASE, CINAHL, and Web of Science from database inception to June 2026. Two reviewers were involved in the search, extraction, quality assessment and synthesis process. A total of 6831 articles were screened, and 36 studies were included, most of which were conducted in high‐income countries. One hundred and thirty extracted findings were used to form 10 categories, which were developed into four synthesised findings: (1) society and culture impact acceptability and visibility of breastfeeding beyond 1 year; (2) structural and institutional constraints undermine breastfeeding beyond 1 year; (3) social networks and peer support influence breastfeeding beyond 1 year; and (4) breastfeeding beyond 1 year involves an interplay of bonding, empowerment, challenges, and adaptation. This review highlights the complex and often challenging contexts women face when breastfeeding beyond 1 year. Multi‐level efforts to normalise and validate this practice are essential to better align with global health guidance. Future research should explore experiences of breastfeeding beyond 1 year in countries where it is more commonly practised.

## Summary

- Social stigma shapes how women manage breastfeeding beyond 1 year.
- Institutional, workplace and healthcare barriers often undermine breastfeeding beyond 1 year, highlighting the need for healthcare professionals to be equipped to provide evidence‐based support in line with international recommendations.
- Peer support networks provide crucial validation and practical support for breastfeeding beyond infancy.
- Consistent public health messaging is needed to normalise breastfeeding beyond 1 year and reduce stigma.
- Future research should examine experiences across diverse cultural settings and at different child ages, particularly beyond 2 years, and improve methodological transparency to strengthen the evidence base.

## 1 Introduction

The World Health Organisation (WHO) recommends ‘*continued breastfeeding up to 2 years of age or beyond*’ (World Health Organisation n.d.). Continued breastfeeding has been associated with a range of benefits for both mother and infant. A dose‐dependent relationship has been observed between longer breastfeeding duration and reduced risk of childhood obesity (Horta et al. 2023; Harder et al. 2005) and type 2 diabetes in later life (Hummel et al. 2021; Alotiby 2023). For the mothers, breastfeeding beyond 1 year has been linked with reduced risk of breast and ovarian cancer (Chowdhury et al. 2015). In addition to health‐related outcomes, breastfeeding is also promoted as an environmentally sustainable practice, with a significantly lower carbon footprint than human milk substitutes (Karlsson et al. 2019). Furthermore, breastfeeding may also contribute to emotional closeness and bonding, which can foster maternal well‐being (Modak et al. 2023).

Rates of breastfeeding beyond 1 year tend to be lower in high‐income countries compared to those in middle‐ and low‐income countries (Victora et al. 2016). However, data on breastfeeding beyond 1 year are not routinely collected in many high‐income countries, limiting understanding of the prevalence of this practice. These disparities have been attributed to a range of structural, commercial and cultural barriers, including early breastfeeding difficulties, restrictive maternity leave policies and limited workplace accommodations (Steurer 2017; Pérez‐Escamilla et al. 2023; Gianni et al. 2019), insufficient healthcare and community support (Keitt et al. 2018; Zhuang et al. 2020), and social norms that often stigmatise breastfeeding beyond 1 year (Tomori et al. 2016).

A range of terms are used to describe breastfeeding beyond 1 year (Dowling and Pontin 2017), including *extended breastfeeding*, *continuous breastfeeding*, *long‐term breastfeeding*, and *breastfeeding beyond infancy*. In this review, the term *breastfeeding beyond 1 year* has been adopted, as it offers a neutral, descriptive framing without implying normative connotations regarding breastfeeding duration.

Existing breastfeeding research predominantly focuses on the early postpartum period, particularly initiation, duration and early discontinuation (Piovanetti 2001). In contrast, less attention has been paid to the experiences of breastfeeding beyond 1 year (Ojantausta et al. 2023). Nevertheless, such experiences are important to understand the broader social, emotional, and cultural dynamics of breastfeeding. Indeed, understanding these experiences is essential to providing better support for women who choose to breastfeed beyond the first year, and for improving public health policies that maintain breastfeeding practices.

A systematic aggregation of qualitative findings on the experiences of women who breastfeed beyond 1 year can contribute to a more holistic understanding of their motivations, challenges, and the forms of resources required to continue breastfeeding. To date, no systematic reviews of qualitative studies focusing on breastfeeding beyond 1 year have been published. Previous reviews exploring breastfeeding beyond 1 year have concentrated on competencies of healthcare professionals (Ojantausta et al. 2023), health impacts upon infants (Lackey et al. 2021) or quantitative determinants associated with duration (Santana et al. 2018).

Accordingly, this systematic review aimed to investigate the lived experiences of women who breastfeed beyond 1 year, using the Joanna Briggs Institute (JBI) meta‐aggregation approach to synthesising qualitative research (Porritt et al. 2024). Meta‐aggregation offers a rigorous and structured approach to integrating qualitative findings, with the aims of informing practice, policy, and future research (Porritt et al. 2024). The review was guided by the following research question: what are the experiences of women who breastfeed beyond 1 year?

## 2 Methods

This review was undertaken using guidelines of the JBI on qualitative evidence synthesis and the meta‐aggregation approach (Porritt et al. 2024). The protocol for this study is registered with the PROSPERO database (ID: CRD420251052793) and we report our work using the Enhancing transparency in reporting the synthesis of qualitative research (ENTREQ) statement (Tong et al. 2012). The described methods below differed slightly from the protocol in the data extraction process. Instead of two reviewers independently extracting the study findings as planned, one reviewer undertook this, and this was subsequently cross‐checked by a second reviewer, for efficiency purposes. Reflexivity was considered for those authors directly involved in study selection, data extraction, and the generation of themes (see Supporting Information).

### 2.1 Search Strategy

In consultation with a medical librarian, a search strategy was planned and executed over four databases: Ovid MEDLINE, Ovid EMBASE, CINAHL and Web of Science (Supporting Information). Searches of grey literature were not undertaken to maintain methodological consistency and ensure the reliability of the evidence included. Only English language articles were included, and databases were searched from inception until the time searches were completed in June 2025. The searches were subsequently updated in June 2026 and any newly identified eligible studies were incorporated into the review.

Hand searches were also performed by reviewing the references of articles included in the systematic review. In addition, the Google Scholar ‘cited by’ feature was utilised to identify any relevant articles that had cited already included works.

### 2.2 Eligibility Criteria

In line with JBI recommendations, eligibility criteria were based on the PICo framework: *Population/Participants* (P); *Phenomenon of interest* (I); *Context* (Co) (Table 1). Studies that combined the experiences of women who breastfed for more than 1 year with those who breastfed for a shorter duration were included only if the experiences of breastfeeding beyond 1 year were clearly and exclusively identifiable, and only these specific experiences were extracted from the study. Studies from all geographic settings were eligible for inclusion to enable a comprehensive exploration of experiences of breastfeeding beyond 1 year across different cultural and healthcare contexts.

**Table 1:** Inclusion criteria.

Population/participants	Phenomenon of interest	Context
Studies involving a woman or women who have breastfed a child beyond one year, including expression and/or from the breast.	Studies reporting on the lived experiences of women breastfeeding beyond one year.	Studies conducted in any setting or geographic location.

Original peer‐reviewed research with a qualitative design or mixed‐method studies with a qualitative element were deemed eligible for inclusion, in combination with the other criteria outlined. For the mixed‐method studies, only the qualitative data was extracted for analysis. No restrictions were placed on publication date to capture the full range of qualitative study on the topic. Studies published up to the date of the updated searches in June 2026 were eligible for inclusion.

### 2.3 Study Selection

The retrieved citations from the four databases were imported into Endnote Online and duplicates were removed. Two reviewers (TC and NAGA) undertook title and abstract screening and full‐text review, according to the eligibility criteria. Discrepancies in study selection were resolved through discussion, with the involvement of a third reviewer (NM) if necessary.

### 2.4 Quality Appraisal of Included Studies

The methodological quality of the studies was determined by two reviewers (TC and NAGA). Reviewers utilised the JBI Critical Appraisal Checklist for Qualitative Research (Lockwood et al. 2015), which comprises 10 items to evaluate research methodology and key aspects of study reporting. All studies, regardless of their methodological quality scores, were included in data extraction and synthesis.

### 2.5 Data Extraction

Data extraction was conducted in two phases by one reviewer (TC). The first phase involved extracting study characteristics, including: first author and year of publication, study country, qualitative methodological approach, data collection procedures, phenomenon of interest, participant details and sampling technique, and data analysis method into a standardised extraction form (Supporting Information).

The second phase encompassed extracting the study findings relevant to this review's question, again into a standardised extraction form (Supporting Information). Findings were typically represented as author‐identified themes or sub‐themes, with an accompanying illustration, which included a participant quotation, fieldwork observation, or other supporting source data. A finding was then assigned a level of credibility—‘Unequivocal’, ‘Credible’, or ‘Not Supported’— depending on the degree of support each illustration offers the specific finding it is associated with (Porritt et al. 2024). These credibility classifications are defined within the JBI meta‐aggregation methodology, whereby unequivocal findings are supported by a detailed and clearly linked illustration that strongly reflected the authors' interpretation. Credible findings were supported by an illustration, although the connection to the interpretation was less explicit or richly described. Not supported findings lacked an accompanying illustration or sufficient supporting data to substantiate the interpretation (Porritt et al. 2024).

All extracted data were cross‐checked by a second reviewer (NAGA) against the original papers to ensure accuracy and completeness. Any discrepancies identified during this process were discussed between the two reviewers and resolved by consensus, with the involvement of a third reviewer (NM), if necessary.

### 2.6 Data Synthesis

Only findings deemed unequivocal or credible were included in the JBI meta‐aggregation process.

This firstly involved grouping similar findings into a category, with this defined as ‘*a brief description of a key concept arising from the aggregation of two or more like findings, based on the similarity of meaning and representation of the phenomenon of interest’* (Porritt et al. 2024). Categories, with an accompanying descriptive term, were developed through an iterative process of reading and re‐reading the findings. Individual findings could contribute to more than one category if appropriate.

Following this, two or more categories were used to develop synthesised findings. These were represented by a descriptive statement and grounded in the data and clearly linked back to the review's aim.

The development of categories and synthesised findings was undertaken by two reviewers (TC and NAGA) through a process of discussion and refinement. Consensus was reached through this process, with a third reviewer (NM) available to resolve disagreements if necessary.

### 2.7 Confidence of Findings

The JBI ConQual process was employed to grade confidence in the synthesised findings (Munn et al. 2014). All synthesised findings started at a high score and were subsequently assessed for dependability and credibility. Dependability was assessed using five items from the JBI Critical Appraisal Checklist across the primary included studies, whilst credibility was determined from the level of unequivocal findings that made up the synthesised findings. If there was an issue with either, dependability could be downgraded by up to two levels and credibility by up to four levels, which then gave rise to a ConQual score.

## 3 Results

### 3.1 Search Results

**Figure 1:**
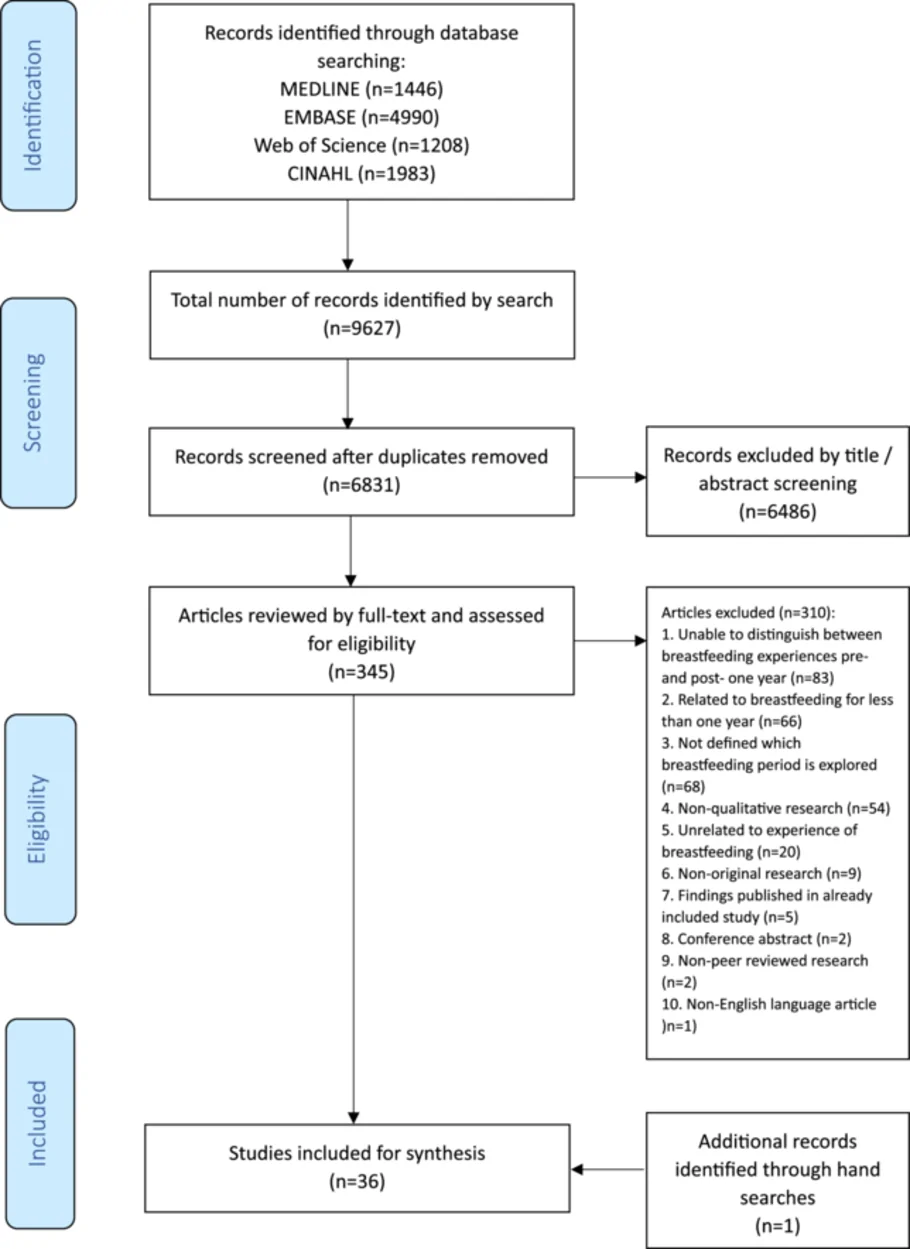
PRISMA flow diagram of study selection.

A total of 6831 articles were screened for eligibility, and 345 were selected for full‐text review (Figure 1). Reasons for studies to be excluded after full‐text review included lack of clarity regarding the breastfeeding period, focus on breastfeeding for less than 1 year, use of non‐qualitative or non‐original research, and duplication of findings. One study was identified from the ‘Cited by’ feature on Google Scholar. A total of 36 studies were ultimately included in the analysis.

### 3.2 Characteristics of Included Studies

The 36 included studies were published between 1990 and 2025, and each employed a qualitative methodology or included a qualitative component (Supporting Information). Most included studies were conducted in high‐income countries, with a smaller number undertaken in lower‐ and upper‐middle‐income settings, alongside studies conducted in socioeconomically diverse contexts such as refugee populations.

The studies employed a range of qualitative approaches, including grounded theory, ethnography, phenomenology, and exploratory designs. Semi‐structured interviews were the most common method of data collection (Dowling and Pontin 2017; Wrigley and Hutchinson 1990; Guyer et al. 2012; Demétrio et al. 2013; Olanders 2013; Cunniff and Spatz 2017; Faircloth 2017; Keevash et al. 2018; Newman and Williamson 2018; Skelton et al. 2018; Martínez‐Poblete and Ossa 2020; Thompson et al. 2020; Buckley 1992; Iglesias‐Rosado and Leon‐Larios 2021; Jackson and Hallam 2021; Burton et al. 2022; Acquaye and Spatz 2023; Goulden et al. 2023; Paul et al. 2024; Marquis et al. 1998; Nelson and Sethi 2005; Gatrell 2007; van den Berg and Ball 2008; Brown et al. 2011; Demirtas et al. 2012; Stearns 2011), though some studies also used written narratives (Säilävaara 2020), focus groups (Skelton et al. 2018; Augusto et al. 2023; White et al. 2012), surveys with open‐ended responses (Brown et al. 2011; Dowling and Brown 2013; Manhire et al. 2007; Gribble 2008; Froh and Spatz 2016; Yate 2017) and photo‐elicitation (Burton et al. 2022), or participant observation (Dowling and Pontin 2017; Faircloth 2017; Buckley 1992; Demirtas et al. 2012; Dowling and Brown 2013). Sample sizes varied widely, from fewer than 10 participants to over 1,300 in one study that included both qualitative and quantitative elements (Dowling and Brown 2013). The studies were conducted in the UK (11), US (7), Canada (2), multiple countries (3), and other single countries (one each in 13 other countries, including 6 in Europe, 3 in South America).

Participants in the included studies were typically mothers with experience of breastfeeding beyond 1 year, though in some cases the original study samples also included those breastfeeding for shorter durations. Only data relevant to breastfeeding beyond 1 year were extracted for the meta‐aggregation. Recruitment occurred through breastfeeding support groups (Dowling and Pontin 2017; Faircloth 2017), social media platforms (Burton et al. 2022; Augusto et al. 2023), online forums (Thompson et al. 2020; Säilävaara 2020), and parenting networks. Other studies recruited participants through healthcare clinics (Demirtas et al. 2012), community organisations (Brown et al. 2011), childcare settings (Goulden et al. 2023), posters or advertisements (Newman and Williamson 2018; Gribble 2008), and survey follow‐up sampling (Keevash et al. 2018)

The studies examined a range of dimensions relating to women's experiences of breastfeeding beyond 1 year, including personal beliefs, social responses, and challenges encountered.

### 3.3 Quality Appraisal of Included Studies

Across the 36 included studies, methodological quality was generally moderate to high, as evaluated using the JBI Critical Appraisal Checklist for Qualitative Research (Lockwood et al. 2015) (Supporting Information). Most studies met key criteria related to methodological congruence, with alignment between their research methodology, objectives, data collection methods, and interpretation of findings. All studies adequately represented participants' voices and provided conclusions that clearly flowed from the data analysis, with 27 (75%) studies reporting ethical approval was obtained for their research. Researcher reflexivity was less frequently addressed, with 11 (31%) studies locating the researcher culturally or theoretically and nine (25%) explicitly addressing the researcher's influence on the research process.

### 3.4 Meta‐Aggregation

A total of 141 findings were extracted from the included studies, with an accompanying illustration (Supporting Information). Fifty‐one were unequivocal, 79 were credible and 11 were not supported findings, meaning 130 were ultimately eligible for the meta‐aggregation. These findings were grouped into 10 categories (Supporting Information) and could appear in more than one category where appropriate. In turn, these were further gathered into four synthesised findings (Table 2) and are outlined below, each with an illustrative quote from one of the included papers.

**Table 2:** Categories (with associated numbers of findings) and corresponding synthesised findings developed through the JBI meta‐aggregation process.

Categories (number in brackets)	Synthesised finding
1. Social judgement, stigma and cultural perceptions (total findings = 27) 2. Public visibility and self‐protection strategies (total findings = 12)	1. Society and culture impact acceptability and visibility of breastfeeding beyond 1 year
3. Influence of the workplace (total findings = 5) 4. Healthcare professional support (total findings = 8)	2. Structural and institutional constraints undermine breastfeeding beyond 1 year
5. Role of breastfeeding organisations and peer support (total findings = 11) 6. Influence of partners, family and friends (total findings = 17)	3. Social networks and peer support influence breastfeeding beyond 1 year
7. Breastfeeding as a parenting tool and emotional bond (total findings = 15) 8. Identity and empowerment (total findings = 13) 9. Physical and emotional challenges (total findings = 15) 10. Motivation and adaptation (total findings = 24)	4. Breastfeeding beyond 1 year involves an interplay of bonding, empowerment, challenges, and adaptation

#### 3.4.1 Synthesised Finding 1—Society and Culture Impact Acceptability and Visibility of Breastfeeding Beyond 1 Year

I just think that culturally there's such a negative association with it [breastfeeding beyond one year] that it does massively impact on the amount of people who do it for longer than six months, so it's just total lack of education, like, for me, I didn't even know you did feed after six months.

Participant quote from (Paul et al. 2024)

This synthesised finding comprised two categories: *Social judgement, stigma and cultural perceptions* and *Public visibility and self‐protection strategies*. It captured a common theme amongst the included studies, whereby women who breastfed beyond 1 year frequently experienced a social and cultural environment that often positioned their practices as abnormal, shameful, or inappropriate (Dowling and Pontin 2017; Wrigley and Hutchinson 1990; Olanders 2013; Newman and Williamson 2018; Skelton et al. 2018; Brown et al. 2011; Stearns 2011; Dowling and Brown 2013; Manhire et al. 2007; Morgan et al. 2026). Across the studies, participants described experiences ranging from subtle disapproval to overt criticism from sources including family, friends and wider public (Newman and Williamson 2018; Buckley 1992; Jackson and Hallam 2021; Burton et al. 2022; Acquaye and Spatz 2023; Paul et al. 2024; Stearns 2011), as well as from media portrayals of breastfeeding beyond 1 year (Newman and Williamson 2018). This could lead to women experiencing pressure to stop breastfeeding (Acquaye and Spatz 2023; Augusto et al. 2023). Several findings also highlighted how breastfeeding in the early months was viewed as more socially acceptable, while breastfeeding beyond 1 year was increasingly seen as excessive or strange (Keevash et al. 2018; Thompson et al. 2020; Jackson and Hallam 2021; Dowling and Brown 2013; Morgan et al. 2026).

As a consequence of these social and cultural pressures, women frequently developed strategies to reduce the visibility of breastfeeding beyond 1 year. This included concealing breastfeeding (Dowling and Pontin 2017; Newman and Williamson 2018; Stearns 2011), using code words to refer to breastfeeding (Wrigley and Hutchinson 1990; Stearns 2011) or not disclosing their practice at all (Wrigley and Hutchinson 1990; Thompson et al. 2020). These approaches often reflected a broader need for women to self‐protect their practice of breastfeeding beyond 1 year from prevailing social and cultural expectations (Olanders 2013; Thompson et al. 2020; Morgan et al. 2026).

Although negative social and cultural influences predominated, some studies highlighted contexts in which breastfeeding beyond 1 year was positively supported. For example, a study from Türkiye highlighted how the influence of religion could encourage a mother to breastfeed beyond 1 year (Demirtas et al. 2012), whilst a study in Chile highlighted how local cultural practices positively shaped breastfeeding beyond 1 year (Martínez‐Poblete and Ossa 2020).

#### 3.4.2 Synthesised Finding 2—Structural and Institutional Constraints Undermine Breastfeeding Beyond 1 Year

The consultant made some comments about how I should be considering weaning and not feeding my baby anymore because of her age… he told me there were no benefits to breastfeeding beyond two, and he told me that breastfeeding hinders children's development.

Participant quote from (Thompson et al. 2020)

The next synthesised finding involved the two categories: *Influence of the workplace* and *Healthcare professional support*. It illustrates how women encountered barriers within institutional settings, particularly healthcare services and workplaces, that often failed to support breastfeeding beyond 1 year.

Within healthcare settings, women frequently described negative comments from members of healthcare professional institutions, particularly doctors, around breastfeeding beyond 1 year (Thompson et al. 2020; Jackson and Hallam 2021; Acquaye and Spatz 2023; Dowling and Brown 2013; Morgan et al. 2026). This tended to pressure women into stopping, rather than accommodating or validating breastfeeding beyond 1 year. While barriers were more commonly reported, examples of supportive healthcare professionals and employers were also identified. Two studies described positive experiences from public health nurses and dietitians as a source of support for this practice (Demétrio et al. 2013; Paul et al. 2024).

Similarly, workplaces could also create environments where breastfeeding beyond 1 year was deterred. Whilst some employers could be supportive (Froh and Spatz 2016), it was reported that this varied, with individuals rather than any policies in the workplace being the deciding factor in how facilitative a place was (Paul et al. 2024). In one study, a feeling of being compelled to hide breastfeeding practices from an employer was described (Gatrell 2007), whilst others faced structural challenges in balancing work responsibilities and breastfeeding (Gatrell 2007; Augusto et al. 2023). This complicated the experience for mothers attempting to navigate both professional or paid work and parenting roles.

#### 3.4.3 Synthesised Finding 3—Social Networks and Peer Support Influence Breastfeeding Beyond 1 Year

It's completely normal, completely natural, and I think having seen people feeding children that were eighteen months, two years, it made me realise it's not weird, it's completely normal. It had a major influence.

Participant quote from (Jackson and Hallam 2021)

The third synthesised finding consisted of the categories: *Role of breastfeeding organisations and peer support* and *Influence of partners, family and friends*. Women frequently highlighted instances of supportive social environments, particularly peer groups, as validating and reassuring in their decision to breastfeed beyond 1 year (Thompson et al. 2020; Burton et al. 2022; Acquaye and Spatz 2023). Online breastfeeding groups, linked to social media, and established organisations such as La Leche League were identified as spaces where this practice was normalised and encouraged (Dowling and Pontin 2017; Newman and Williamson 2018; Buckley 1992; Jackson and Hallam 2021; Gribble 2008; Morgan et al. 2026). Women described learning from others who were breastfeeding older children or feeling inspired by witnessing breastfeeding beyond 1 year in action in these settings (Demétrio et al. 2013; Jackson and Hallam 2021; Paul et al. 2024). These experiences often helped to counter negative messages encountered in more mainstream contexts (Jackson and Hallam 2021; Augusto et al. 2023).

The responses from traditional social networks, including partners, family members, and friends, tended to be mixed (Newman and Williamson 2018; Acquaye and Spatz 2023; Stearns 2011; Augusto et al. 2023; Morgan et al. 2026). Whilst there were examples of encouragement from these close contacts (Jackson and Hallam 2021; Paul et al. 2024), some women also described being challenged or criticised, and in some cases, pressured to stop breastfeeding (Jackson and Hallam 2021; Augusto et al. 2023; Froh and Spatz 2016). Partners were identified as a key influence, with their views playing a pivotal role in either supporting or undermining breastfeeding beyond 1 year (Newman and Williamson 2018; Martínez‐Poblete and Ossa 2020; Buckley 1992; Iglesias‐Rosado and Leon‐Larios 2021; Paul et al. 2024; Augusto et al. 2023; Morgan et al. 2026).

#### 3.4.4 Synthesised Finding 4—Breastfeeding Beyond 1 Year Involves an Interplay of Bonding, Empowerment, Challenges, and Adaptation

With my first daughter I really found breastfeeding harder towards the end, wanted my body back and in the end we ended the feeding probably a bit to abruptly, (at age 2 years and 1 month), and I do regret that in some ways. This time I've decided to go on for a bit longer, although at some point I would like to start gently weaning. I would love for things to come to a natural end but I am not sure I can feed for another 18 months if that's how long it will take.

Participant quote from (Burton et al. 2022)

The final synthesised finding contained four categories: *Breastfeeding as a parenting tool and emotional bond, Identity, and empowerment, Physical and emotional challenges*, and *Motivation and adaptation*. This finding featured more descriptions of individual choice and agency than the other three. Women described breastfeeding beyond 1 year as a responsive parenting tool, useful for soothing and meeting their child's needs (Wrigley and Hutchinson 1990; Thompson et al. 2020; Buckley 1992; Burton et al. 2022; White et al. 2012; Morgan et al. 2026). They emphasised the emotional bond it fostered (Wrigley and Hutchinson 1990; Demétrio et al. 2013; Burton et al. 2022; Morgan et al. 2026), along with its practical value and perceived health benefits (Martínez‐Poblete and Ossa 2020; Thompson et al. 2020; Paul et al. 2024; Morgan et al. 2026).

For many, breastfeeding also contributed to their maternal identity (Wrigley and Hutchinson 1990; Olanders 2013; Morgan et al. 2026) and shaped their relationship with their bodies (Säilävaara 2020; Augusto et al. 2023). Some described feeling more confident (Säilävaara 2020; Augusto et al. 2023), while tension around partner intimacy was also noted (Augusto et al. 2023). Breastfeeding was described as feeling “natural” and “normal” (Olanders 2013; Faircloth 2017; Thompson et al. 2020), and for some, continuing beyond 1 year brought a sense of achievement or empowerment (Thompson et al. 2020; Paul et al. 2024; Säilävaara 2020).

However, accompanying these benefits, significant physical and emotional demands were also discussed in relation to this practice (Buckley 1992; Augusto et al. 2023). Mothers described feelings of exhaustion and sleep disruption (Cunniff and Spatz 2017; Paul et al. 2024; Säilävaara 2020). Additionally, it could represent a time‐demanding practice and a requirement to share one's body with their child (Wrigley and Hutchinson 1990; Burton et al. 2022; Marquis et al. 1998; Morgan et al. 2026). Breastfeeding beyond 1 year whilst pregnant was also discussed, including the challenges with this (van den Berg and Ball 2008), as well as its potential impact on family planning and fertility (Cunniff and Spatz 2017). Two articles also discussed breastfeeding aversion where women experienced feelings of discomfort and agitation whilst breastfeeding, but nevertheless continuing to do so (Yate 2017; Morgan et al. 2026).

These accounts highlight the adaptation involved in sustaining breastfeeding beyond 1 year, from adjusting expectations about duration, to changing sleeping arrangements (such as co‐sleeping) to facilitate night breastfeeding (Thompson et al. 2020; Buckley 1992; Paul et al. 2024; Gribble 2008). Knowledge of breastfeeding's benefits gave women purpose (Paul et al. 2024; Gribble 2008), and many described a child‐led approach as central to their motivation (Dowling and Pontin 2017; Wrigley and Hutchinson 1990; Olanders 2013; Faircloth 2017; Thompson et al. 2020; Morgan et al. 2026; Lal‐Kumar et al. 2026). Still, for some, the process of stopping breastfeeding raised internal conflict, particularly around whether the decision to stop should rest with the mother or the child (Guyer et al. 2012; Burton et al. 2022; Acquaye and Spatz 2023; Goulden et al. 2023; Marquis et al. 1998; Nelson and Sethi 2005; Morgan et al. 2026).

### 3.5 ConQual Assessment

The overall ConQual scores for all of the four synthesised findings were rated as Low, reflecting limitations in their dependability and credibility (Table 3), with a level of caution therefore recommended in their interpretation. This related to issues around methodological reporting, particularly around researcher reflexivity, and the presence of findings deemed only credible rather than unequivocal. Nevertheless, despite the low ConQual ratings, similar themes were consistently identified across a large number of studies conducted in diverse settings, suggesting a notable degree of consistency in the qualitative evidence base.

**Table 3:** ConQual summary of findings.

Synthesised finding	Dependability	Credibility	ConQual score
1. Society and culture impact acceptability and visibility of breastfeeding beyond 1 year	Downgrade 1 level^a^	Downgrade 1 level^b^	Low
2. Structural and institutional constraints undermine breastfeeding beyond 1 year	Downgrade 1 level^a^	Downgrade 1 level^b^	Low
3. Social networks and peer support influence breastfeeding beyond 1 year	Downgrade 1 level^a^	Downgrade 1 level^b^	Low
4. Breastfeeding beyond 1 year involves an interplay of bonding, empowerment, challenges, and adaptation	Downgrade 1 level^a^	Downgrade 1 level^b^	Low

a Dependability downgraded due to inconsistent reporting of researcher reflexivity across included studies.

b Credibility downgraded due to a mix of unequivocal (U) and credible (C) findings contributing to each synthesised finding.

## 4 Discussion

### 4.1 Principal Findings

This review synthesised qualitative research exploring the lived experiences of women who breastfed beyond 1 year. The research predominantly occurred in high‐income countries where rates of breastfeeding beyond 1 year have tended to be lower (Victora et al. 2016). The included studies highlighted the multifaceted and often challenging contexts in which breastfeeding beyond 1 year occurs. The findings revealed that this practice is frequently met with social stigma and cultural disapproval, leading many women to reduce the visibility of breastfeeding and having to manage criticism from family, friends and the wider public. These negative evaluations extended into professional environments, with workplace constraints and unsupportive healthcare professionals further undermining the ability to breastfeed beyond 1 year.

In the absence of consistent support from traditional networks, peer groups, social media communities, and breastfeeding organisations played a critical role in providing validation and encouragement. These alternative support systems often enabled and inspired women to continue the practice despite external pressures.

The review also illustrated the profound impacts breastfeeding beyond 1 year has upon women. It was viewed as an important parenting tool and a source of emotional closeness with the child. Furthermore, the practice could shape elements of their maternal identity, from attitudes towards their body to feelings of empowerment from being able to breastfeed beyond 1 year. This provided motivation, alongside the need for regular adjustment to continue breastfeeding. At the same time, the experience could be physically and emotionally demanding, with women describing internal conflicts involved in the timing and process of stopping breastfeeding.

### 4.2 Comparison to Existing Literature

The findings of this meta‐aggregation align with key themes identified in recent qualitative and systematic reviews on breastfeeding more broadly, as well as breastfeeding beyond 1 year. Societal and cultural factors continue to shape the acceptability of breastfeeding practices, as demonstrated in syntheses examining breastfeeding in public (Grant et al. 2022; Hauck et al. 2021). These reviews highlight how pervasive stigma and the sexualisation of breastfeeding contribute to women reducing the visibility of their breastfeeding practices.

Structural and institutional barriers to breastfeeding identified in this review are similarly reflected in the literature. For example, a review of healthcare professionals' competencies in supporting breastfeeding beyond 1 year reported attitudes of ambivalence or hostility and insufficient knowledge (Ojantausta et al. 2023). Whilst a scoping review of women's experience of breastfeeding more generally identified return to employment as a significant challenge to continue breastfeeding (Goulden et al. 2023). While noting that these same social and structural influences are present in the experience of those breastfeeding infants, the evidence in this review highlighted these features appear to be more strongly experienced when breastfeeding beyond 1 year.

The value of social networks and peer support, as emphasised by participants in this synthesis, is also consistent with the existing literature. A Cochrane review demonstrated the effectiveness of lay support in promoting breastfeeding (Sikorski et al. 2004), while social media groups have been identified as important facilitators of breastfeeding encouragement (Morse and Brown 2022). The role of partners, family members, and friends in influencing breastfeeding decisions has also been consistently highlighted across studies (Grant et al. 2022; Hauck et al. 2021; Negin et al. 2016; Ogbo et al. 2020; Baranowski et al. 1983), suggesting that positive social influences can help mitigate the effects of wider cultural stigma.

Previous literature has also linked breastfeeding to maternal sleep patterns and infant attachment outcomes (Manková et al. 2023; Baddock et al. 2019; Linde et al. 2020), and acknowledged the emotional complexity surrounding breastfeeding (Peñacoba and Catala 2019; Burns et al. 2010), corresponding with the final theme of this synthesis.

### 4.3 Strengths and Limitations

The review utilised a comprehensive search strategy that included both electronic databases and manual searches, with no restrictions on publication date. Two reviewers were involved in the study selection, quality appraisal, and data extraction process, enhancing the reliability and rigour of the study findings. The review is also novel in its specific focus on synthesising women's experiences of breastfeeding beyond 1 year, filling a gap in the existing literature.

However, several limitations should be acknowledged. First, the majority of included studies were conducted in high‐income countries, which may limit the global applicability of the findings. Social, cultural, and structural influences on breastfeeding beyond 1 year vary considerably across settings, including differences in breastfeeding norms, workplace expectations, and societal attitudes. This may partly reflect differences in research priorities, with breastfeeding beyond 1 year potentially less likely to be studied as a distinct phenomenon in settings where it is more common and socially accepted. Consequently, experiences from countries where longer breastfeeding durations are normative may be underrepresented within the evidence base.

Secondly, many studies recruited participants through breastfeeding support groups, online forums, social media platforms, or breastfeeding‐focused networks. As a result, women who were more engaged with breastfeeding communities may have been overrepresented within the evidence base, potentially influencing the prominence of themes relating to peer support and breastfeeding continuation.

Thirdly, grey literature was not included, which could have enabled the capture of a broader range of findings, and only English‐language articles were considered, which may have introduced language bias. Finally, the included studies spanned more than three decades, during which breastfeeding practices, policies, workplace norms, and social attitudes may have evolved. Consequently, some findings may not be fully transferable to contemporary contexts. However, similar themes were identified across both older and more recent studies, suggesting a degree of consistency in women's experiences over time.

### 4.4 Implications for Policy, Practice and Future Research

In accordance with the JBI approach (Porritt et al. 2024), practice recommendations have been assigned a Grade B, reflecting a weak recommendation based on the low confidence in the synthesised findings. Nevertheless, the review identifies actionable areas to improve the experience of women breastfeeding beyond 1 year.

Healthcare professionals should be better equipped to validate and support breastfeeding beyond 1 year. Incorporating specific education on this practice into general breastfeeding training would help align practice with the WHO's recommendation to continue breastfeeding up to 2 years and beyond (World Health Organisation n.d.). Social support was a recurring theme in the review, with peer networks and breastfeeding organisations often providing the primary source of encouragement. As such, rather than relying solely on healthcare‐based interventions, a more community‐orientated approach may be warranted. This may include strengthening peer support networks and developing more collaborative partnerships between healthcare services and established community breastfeeding organisations.

Workplaces are instrumental in facilitating breastfeeding beyond 1 year, as demonstrated by research highlighting the impact of supportive initiatives (Vilar‐Compte et al. 2021). Creating breastfeeding‐friendly environments, through flexible policies and supportive infrastructure, can reduce pressure on women to choose between employment and continued breastfeeding (Brown 2017). Similarly, the media, which has often portrayed breastfeeding negatively or in sexualised terms (Grant 2016; Foss 2013; Henderson et al. 2000), could contribute to normalising breastfeeding beyond 1 year by improving its visibility.

At the policy level, national health strategies in high‐income countries should explicitly include breastfeeding beyond 1 year within public health frameworks. Consistent messaging across services can help reduce cultural stigma and promote supportive environments. Collectively, these recommendations are consistent with the Becoming Breastfeeding Friendly framework (Pérez‐Escamilla et al. 2018), which highlights the importance of coordinated action across multiple sectors to create enabling environments for breastfeeding.

Future research should address the geographic and cultural limitations of the current evidence base. Studies from countries where breastfeeding beyond 1 year is more common could provide insights into social norms, caregiving practices, and health system supports that sustain breastfeeding. Further research is also needed on women's experiences of stopping breastfeeding, particularly to inform guidance that is emotionally sensitive and responsive to women's needs. Future qualitative studies should improve methodological transparency and reflexivity to enhance dependability. Finally, additional research could also explore how experiences vary according to the age of the child, particularly among women breastfeeding beyond 2 years, as many studies considered breastfeeding beyond 1 year as a single category despite potentially differing social and practical experiences.

## 5 Conclusion

This qualitative systematic review and meta‐aggregation has provided important insights into women's experiences of breastfeeding beyond 1 year. Across the included studies, women described navigating social judgement, institutional pressures, and a frequent lack of traditional supports, highlighting a mismatch between lived experience and global health guidance. Breastfeeding for this duration was shown to have a profound impact on women, marked by both challenge and adaptation, but also by experiences of empowerment, bonding, and personal fulfilment.

Efforts to reduce stigma around breastfeeding for this duration are needed, whilst existing social networks that encourage breastfeeding beyond 1 year should be strengthened. Furthermore, barriers and negative attitudes encountered from interactions with healthcare professionals and workplace environments need to be overcome to better validate and facilitate this practice.

Future research would benefit from exploring experiences of breastfeeding beyond 1 year in more culturally diverse settings and investigating processes around discontinuing breastfeeding beyond 1 year.

## Author Contributions

Conceptualisation: Thomas Cronin. Formal analysis: Thomas Cronin, Noor Ainah Gulzar Ahmad. Funding acquisition: Noor Ainah Gulzar Ahmad, Noel McCarthy. Investigation: Thomas Cronin, Noor Ainah Gulzar Ahmad. Methodology: Thomas Cronin, Noel McCarthy. Project administration: Thomas Cronin. Supervision: Noel McCarthy. Writing – original draft preparation: Thomas Cronin. Writing – review and editing: Noor Ainah Gulzar Ahmad, Noel McCarthy.

## Conflicts of Interest

The authors declare no conflicts of interest.

## Supporting information


Supporting File

## Data Availability

The data that support the findings of this study are available within the article and its Supporting Information. No new datasets were generated during the current study.
