# Supplementary material for: Women's Experiences of Breastfeeding Beyond One Year: A Qualitative Systematic Review
Source: Matern Child Nutr. 2026 Sep 1;22(3):e70242. doi: 10.1111/mcn.70242 (PMC13531375; doi:10.1111/mcn.70242)
Supplement: Supplementary file 1 [file MCN-22-e70242-s001.docx]

**Supplementary Material**

**Search strategy**

**Embase**

1. 'breast feeding'/exp

2. (Breast NEAR/2 feed*):ti,ab,kw

3. ('breast fed' OR breastfeed*):ti,ab,kw

4. 1 OR 2 OR 3

5. (extended OR "long-term" OR "long term" OR continuous OR "beyond infanc*" OR "beyond one year" OR toddler OR "full-term" OR "natural-term"):ti,ab,kw

6. ((twelve OR 12) NEAR/2 month* NEAR/3 (more OR greater OR beyond OR 'in excess' OR 'at least' OR longer OR higher OR older)):ti,ab,kw

7. ('≥ 12 month*' OR 'longer duration' OR 'Older infant*'):ti,ab,kw

8. ((12 OR twelve OR 18 OR eighteen OR 24 OR 'twenty four') NEAR/2 month*):ti,ab,kw

9. Year*:ti,ab,kw

10. 5 OR 6 OR 7 OR 8 OR 9

11. ((('semi structured' OR semistructured OR unstructured OR informal OR 'in depth' OR indepth OR 'face to face' OR structured OR guide) NEAR/3 (interview* OR discussion* OR questionnaire*)):ti,ab) OR 'focus group*':ti,ab OR qualitative:ti,ab OR ethnograph*:ti,ab OR fieldwork:ti,ab OR 'field work':ti,ab OR 'key informant':ti,ab OR 'qualitative research'/de

12. ('experiential account*' OR interview* OR qualitative):ti,ab,kw OR 'health care organization'/exp

13. 11 OR 12

14. 'mother'/exp

15. (Mother OR maternal):ti,ab,kw

16. 14 OR 15

17. 4 AND 10 AND 13 AND 16

**Medline**

1. exp breast feeding/

2. (Breast adj2 feed*).tw.

3. (breast fed OR breastfeed*).tw.

4. or/1-3

5. (extended OR "long-term" OR "long term" OR continuous OR "beyond infanc*" OR "beyond one year" OR toddler OR "full-term" OR "natural-term").tw.

6. ((twelve OR 12) adj2 month* adj3 (more OR greater OR beyond OR "in excess" OR "at least" OR longer OR higher OR older)).tw.

7. ("longer duration" OR Older infant*).tw.

8. ((18 OR eighteen OR 24 OR "twenty four") adj2 month*).tw.

9. Year*.tw.

10. or/5-9

11. exp mothers/

12. (Mother* OR maternal).tw.

13. 11 OR 12

14. (((("semi-structured" OR semistructured OR unstructured OR informal OR "in-depth" OR indepth OR "face-to-face" OR structured OR guide) adj3 (interview* OR discussion* OR questionnaire*))).ti,ab. OR (focus group* OR qualitative OR ethnograph* OR fieldwork OR "field work" OR "key informant" OR "experiential account*").ti,ab. OR interviews as topic/ OR focus groups/ OR narration/ OR qualitative research/)

15. 4 AND 10 AND 13 AND 14

**CINAHL**

1. (MH "Breast Feeding") OR TI ((Breast N2 feed*) OR "breast fed" OR breastfeed*) OR AB ((Breast N2 feed*) OR "breast fed" OR breastfeed*)

2. TI (((Twelve OR 12) N2 month* N3 (more OR greater OR beyond OR "in excess" OR "at least" OR longer OR higher OR older)) OR "longer duration" OR "Older infant*" OR ((12 OR twelve OR 18 OR eighteen OR 24 OR "twenty four") N2 (month* OR postpartum))) OR AB (((twelve OR 12) N2 month* N3 (more OR greater OR beyond OR "in excess" OR "at least" OR longer OR higher OR older)) OR "longer duration" OR "Older infant*" OR ((12 OR twelve OR 18 OR eighteen OR 24 OR "twenty four") N2 (month* OR postpartum)))

3. TI (Year*) OR AB (Year*)

4. TI (extend* OR "long-term" OR "long term" OR continuous OR "beyond infanc*" OR "beyond one year" OR toddler* OR "full-term" OR "natural-term" OR "Older infant*") OR AB (extend* OR "long-term" OR "long term" OR continuous OR "beyond infanc*" OR "beyond one year" OR toddler* OR "full-term" OR "natural-term" OR "Older infant*")

5. S2 OR S3 OR S4

6. (MH "Mothers") OR TI (Mother* OR maternal) OR AB (Mother* OR maternal)

7. (MH "cluster sample+") or TX life experiences or TX human science or TX discourse* analysis or TX narrative analysis or TX lived experience* or TX field research or TX field studies or TX field study or TX giorgi* or TX husserl* or TX merleau ponty* or TX van kaam* or TX van manen* or TX spiegelberg* or TX colaizzi* or TX heidegger* or TX participant observ* or TX data saturat* or TX semiotics or TX heuristic or TX hermeneutic* or TX etic or TX emic or TX focus group* or TX purpos* sampl* or TX constant comparison or TX constant comparative or TX grounded research or TX grounded studies or TX grounded study or TX grounded theor* or TX phenomenol* or TX ethnon* or TX qualitative or (MH "ethnological research") or (MH "ethnography") or (MH "phenomenology") or (MH "focus groups") or (MH "discourse analysis") or (MH "theoretical sample") or (MH "field studies") or (MH "constant comparative method") or (MH "thematic analysis") or (MH "content analysis") or (MH "observational methods+") or (MH "purposive sample") or (MH "qualitative validity+") or (MH "grounded theory") or (MH “action research”) or (MH “naturalistic inquiry”) or (MH "ethnonursing research") or (MH "phenomenological research") or (MH "ethnographic research") or (MH "qualitative studies") or (MH "Interviews+") or (MH "Narratives") or (MH "Videorecording+") or (MH "Audiorecording") or (MH "Historical Records")

8. S1 AND S5 AND S6 AND S7

**Web of Science**

1. (Breast NEAR/2 feed*) OR "breast fed" OR breastfeed*

2. Year* OR ((Twelve OR 12) NEAR/2 month* NEAR/3 (more OR greater OR beyond OR "in excess" OR "at least" OR longer OR higher OR older)) OR "≥ 12 month*" OR "longer duration" OR "Older infant*" OR ((12 OR twelve OR 18 OR eighteen OR 24 OR "twenty four") NEAR/2 (month* OR postpartum)) OR extended OR "long-term" OR "long term" OR continuous OR "beyond infanc*" OR "beyond one year" OR toddler* OR "full-term" OR "natural-term"

3. qualitative OR ethnol* OR ethnog* OR ethnonurs* OR emic OR etic OR leininger OR noblit OR "field note*" OR "field record*" OR fieldnote* OR "field stud*" OR "participant observ*" OR "participant observation*" OR hermeneutic* OR phenomenolog* OR "lived experience*" OR heidegger* OR husserl* OR "merleau-pont*" OR colaizzi OR giorgi OR ricoeur OR spiegelberg OR "van kaam" OR "van manen" OR "grounded theory" OR "constant compar*" OR "theoretical sampl*" OR (glaser AND strauss) OR "content analy*" OR "thematic analy*" OR narrative* OR "unstructured categor*" OR "structured categor*" OR "unstructured interview*" OR "semi-structured interview*" OR "maximum variation*" OR snowball OR audio* OR tape* OR video* OR metasynthes* OR "meta-synthes*" OR metasummar* OR "meta-summar*" OR metastud* OR "meta-stud*" OR "meta-ethnograph*" OR metaethnog* OR "meta-narrative*" OR metanarrat* OR "meta-interpretation*" OR metainterpret* OR "qualitative meta-analy*" OR "qualitative metaanaly*" OR "qualitative metanaly*" OR "purposive sampl*" OR "action research" OR "focus group*" OR photovoice OR "photo voice" OR "mixed method*"

4. Mother* OR maternal

5. #1 AND #2 AND #3 AND #4

**Reflexivity statement of research members**

| **Reflexivity domain*** | **Thomas Cronin (TC)** | **Noor Ainah Gulzar Ahmad (NAGA)** |
| --- | --- | --- |
| *“Previous personal and professional experiences”* | TC is a GP, currently working in public health and undertaking a Master’s in Public Health (MPH). He previously trained in clinical settings and has an interest in health inequalities. His personal experience includes witnessing his partner breastfeed beyond one year, which provided an insight into the benefits and challenges of this practice. | NAGA is 4^th^ year medical student at Trinity College Dublin. She previously studied in University Brunei Darussalam and graduated with a Bachelor of Health Sciences degree. She comes from a community that religiously believes a child should be breastfed for two full years. |
| *“Prestudy beliefs about how things are and what is to be investigated”* | TC acknowledges a belief that breastfeeding beyond one year is not always well supported in healthcare settings and seems underrepresented in public discourse. He also recognises a bias toward prioritising lived experience and peer support in maternal health decisions. | NAGA believes culture plays a significant role in supporting or stigmatising breastfeeding beyond one. She is of the opinion that there is not enough support and encouragement given by the society or healthcare workers to first-time mothers in Ireland who might consider breastfeeding beyond one year. |
| *“Motivation and qualifications for exploration of the field”* | This review was prompted by TC’s MPH studies, with a desire to better understand and highlight women's experiences of breastfeeding beyond one year. He has undertaken prior research using qualitative and evidence synthesis methodologies. His background in general practice has provided some existing understanding of breastfeeding support in a clinical setting, as well as different breastfeeding issues encountered in primary care. | This review was undertaken by NAGA as part of her medical school summer elective. She has previous experience in qualitative literature review, and pre-post study. The topic of breastfeeding was briefly covered in her three years of medical school in Brunei. She has personal experiences of observing her mother breastfeed her younger siblings for full two years. |
| *“Theoretical foundations related to education and interests”* | TC’s perspective is informed by public health and social justice frameworks, including an emphasis on patient-centred care, maternal autonomy, and addressing structural health inequalities. He aimed to conduct the synthesis transparently, while also reflecting on how his views may have shaped interpretation. | NAGA approaches the topic of breastfeeding with an interest in maternal and child health, recognising breastfeeding as both a clinical and public health priority. She considers breastfeeding not only as an individual choice but also as an issue shaped by social, cultural, and structural contexts. She aimed to approach the synthesis with openness, while being mindful that her background and developing clinical identity may lead her to privilege certain interpretations over others. By reflecting on this, she hoped to conduct the review with greater transparency and self-awareness**.** |

*Areas of reflexivity in line with Malterud recommendations [1]

**Characteristics of included studies**

| **Study ID (First author & year published)** | **Country** | **Qualitative methodological approach** | **Data collection method** | **Phenomena of interest** | **Participants, setting and sampling** | **Data analysis** |
| --- | --- | --- | --- | --- | --- | --- |
| Wrigley 1990 [2] | USA | Grounded theory | Semi-structured interviews in participants’ home | Feelings, concerns, conflicts, and relationships of “long-term” breastfeeding mothers. | Purposive sampling of 12 white middle-class mothers who had breastfed > 1 year recruited through a local La Leche League group. | Constant comparative method |
| Buckley 1992 [3] | USA | Unspecified | Semi-structured interviews in participants’ homes. Field notes of observations whilst in the participants’ homes. Mothers were also asked to complete two brief questionnaires on the role of social influence and  social support upon the decision to practice extended breastfeeding. | The beliefs and practices pertinent “extended” breastfeeding mothers | Convenience sampling of 10 mothers who were breastfeeding a child > 1 year attending a Leche League group | Content analysis |
| Marquis 1998 [4] | Peru | Grounded theory | Semi-structured interviews in participants’ homes. | The reversible nature of child-feeding decisions, focusing on breastfeeding, weaning, and re-lactation. | Random sampling of 36 women (data from 32 were included in the analysis, 21 breastfeeding and 11 recently weaned mothers). Participants included women with children aged 12–24 months from a low-income setting in peri-urban shantytown community on the outskirts of Lima, characterised by limited resources, overcrowding and poor sanitation. | Constant comparative method |
| Nelson 2005 [5] | Canada | Grounded theory | Semi-structured interviews generally in participants’ homes, alongside field notes. | The breastfeeding experiences of teenage mothers. | Purposive and theoretical sampling of eight teenage mothers. | Constant comparative method |
| Gatrell 2007 [6] | UK | Unspecified qualitative | Semi-structured interviews (not stated where these took place) | The experiences of professional mothers who were combining paid work with mothering pre-school children. | Snowball sampling of 20 women living across the UK educated to degree level employed in professional or managerial roles who returned to work within a year of childbirth. | Thematic analysis |
| Manhire 2007 [7] | New Zealand | Descriptive | Open-ended responses from a semi-structured questionnaire originally designed for a quantitative study | Breastfeeding experiences of mothers covering initiation, maintenance, challenges, support, and cessation. | A total of 500 women were randomly selected from the study area, with 153 (31% response rate), returning the questionnaire. | Thematic analysis |
| Gribble 2008 [8] | Australia | Unspecified qualitative | Mixed methods, comprising postal questionnaire with quantitative and free text elements | The factors influencing mothers to continue breastfeeding “beyond infancy” and the challenges they face. | A convenience sample was recruited through advertisements, word of mouth, and the Australian Breastfeeding Association of 107 women breastfeeding a child aged two years or older. | Unspecified, “*recurring themes*” |
| van den Burg 2008 [9] | Sri Lanka | Descriptive | Semi-structured interviews generally in participants’ homes | To explore the breastfeeding experiences of women in a culture where “long-term” breastfeeding is the norm. | Purposive and geographic sampling in peri-urban and rural areas of 60 women age 23-45 years. | Thematic analysis |
| Brown 2011 [10] | UK | Unspecified | Mixed methods, comprising a questionnaire and semi-structured interviews | Factors associated with breastfeeding duration in younger mothers aged 24 or younger. | Convenience sampling of participants recruited from day care centres, postnatal groups, and mother and infant groups in the City and County of Swansea, and online parenting forums based in the UK. The questionnaire was completed by 138 mothers (aged ≤24) and interviews with 10 mothers who breastfed beyond 6 months. | Unspecified |
| Demirtas 2011 [11] | Türkiye | Descriptive | Semi-structured interviews in participants’ homes, alongside fieldwork. | Traditional breastfeeding practices among Turkish mothers. | Purposive sampling from records at the local mother and child health centre of 24 mothers aged 15-49 years with infants aged 4-24 months. | Content analysis |
| Stearns 2011 [12] | USA | Unspecified qualitative | Semi-structured interviews generally in participants’ homes. | Mothers' beliefs, experiences, and perceptions of “extended” breastfeeding. | A snowball sampling strategy of 66 mothers in Northern California diverse in class and race from breastfeeding support groups, teen parenting programmes and community organisations. | Inductive approach |
| Guyer 2012 [13] | UK | Phenomenology | Semi-structured interviews in participants’ homes. | Mothers’ experiences of initiating and ceasing, or continuing breastfeeding. | Selective sampling of six mothers (aged 34–42 years) who were currently or had been breastfeeding in the past year. All participants were professional, middleclass women | Interpretative phenomenological analysis |
| White 2012 [14] | Thailand & Myanmar | Unspecified | Mixed-methods, comprising of chart review, focus group with mothers and interviews with midwives. | To explore the views of women and midwives surrounding breastfeeding and swaddling. | Purposive sampling of 50 women who were pregnant and / or had previously breastfed took place an antenatal clinic over nine focus groups. | Unspecified |
| Demétrio 2013 [15] | Brazil | Phenomenology | Semi-structured interviews in participants homes. | Meanings attributed by mothers to breastfeeding during the child’s first two years of life. | Random sampling of urban and rural areas of 15 mothers who breast for more than 24 months. | Thematic analysis |
| Dowling 2013 [16] | UK | Ethnographic | The paper reported the results of two studies which utilised mixed methods. The first comprised of face-to-face and online semi-structured interviews and participant observations in breastfeeding support groups. The second study presented data collected via an open-ended online questionnaire. | **Mothers' experiences and perceptions of “long-term” breastfeeding.** | Purpose and snowball sampling was employed in the studies. Study 1 involved 10 mothers who breastfed for over six months alongside observations of 80+ breastfeeding women from breastfeeding support groups, whilst study 2 consisted of 1,319 participants who were pregnant or with children aged 0-2 years from primarily online platforms. | Thematic analysis |
| Olanders 2013 [17] | Sweden | Unspecified qualitative | Semi-structured interviews in participants’ homes or public places (e.g. café, library) | How mothers who breastfeed preschool children experience being treated by preschool staff. | Participants were recruited via breastfeeding and parenting websites and forums in Sweden and included ten mothers, aged 30–46 (median age 37), who breastfed their children while they attended preschool. | Content analysis |
| Froh 2016 [18] | USA | Descriptive | Secondary analysis of open-ended responses to an online survey. | Experiences with breastfeeding after return to work | A convenience sample of 410 mothers employed in a Philadelphia children’s hospital who provided a free-text response to the original questionnaire. | Content analysis |
| Cunniff 2017 [19] | USA | Unspecified | Face to-face interviews using a topic guide in a location determined by the research participant. | Weaning practices of mothers that choose to breastfeed beyond one year. | Convenience sampling of four mothers recruited through a local breastfeeding support group’s social media site | Unspecified |
| Dowling 2017 [20] | UK | Microethnography | Participant observation in breastfeeding support groups, and face-to-face and online interviews. | The experiences of women who breastfeed “long-term”. | Purposive and snowball sampling methods were employed for the interviews whilst three breastfeeding support groups including La Leche League were observed. Over 80 breastfeeding women participated in some form. Participants varied in socio-economic backgrounds, relationship status and working status. | Thematic analysis |
| Faircloth 2017 [21] | UK & France | Ethnography | Participant observation in breastfeeding support groups, and semi-structured interviews and email surveys. | “Full-term” breastfeeding as part of attachment parenting. | Observation at 18 local breastfeeding support groups, with interviews of 26 mothers attending these groups and 13 of the support group leaders. Additionally, an email survey with 48 women was also conducted. Participants were predominantly white, university-educated and married. | Comparative approach |
| Yate 2017 [22] | India, Iran, UK & USA | Exploratory | Online survey with qualitative component | To explore the experience of breastfeeding aversion and agitation. | Purposive voluntary sampling of 694 breastfeeding women from multiple countries recruited via social media and online parenting groups. | Thematic and content analysis |
| Keevash 2018 [23] | UK | Unspecified | Semi-structured interviews | Experiences and influencing factors affecting continuation or cessation of breastfeeding | Random sampling of 41 women who had participated in an online survey that had been responded to by 1542 participants. | Thematic analysis |
| Newman 2018 [24] | UK | Phenomenology | Semi-structured interviews | Experiences of breastfeeding beyond six months. | Purposive sampling of eight women through posters and online recruitment, taking place in participants’ homes and libraries. Participants were aged 26–36 years, all were heterosexual, in long-term relationships, and had post-compulsory education (six had university degrees). | Interpretative phenomenological analysis |
| Skelton 2018 [25] | USA | Unspecified | Focus groups and semi-structured interviews | Breastfeeding mothers’ use of a social media group and how it shapes their breastfeeding experience, attitudes, knowledge, and outcomes. | Purposeful sampling through snowball design, with recruitment from an online breastfeeding group. Focus groups were conducted with 21 women and interviews were conducted with 12 mothers. | Thematic analysis |
| Martínez-Poblete 2020 [26] | Chile | Grounded theory | Semi-structured interviews | Motivations for “prolonged” breastfeeding. | Theoretical sampling of eight women who breastfed their last child for over two years in a mix of urban and rural settings. | Constant comparative method |
| Säilävaara 2020 [27] | Finland | Exploratory | Written narratives collected via email and online submissions | The embodied experiences of “long-term” breastfeeding mothers. | Convenience and snowball sampling through social media and online forums of 39 mothers. Most participants were university educated, married or in a civil partnership, and had multiple children. | Inductive content analysis |
| Thompson 2020 [28] | UK | Interpretative, exploratory | Semi-structured interviews online and face-to-face (in participants’ homes) | Women's beliefs and motivations regarding breastfeeding “beyond infancy”. | Purposive sampling of 19 women 18+ years who were either currently breastfeeding a child older than one year or had done so within the previous five years. Recruitment was via online breastfeeding support groups. Participants were predominantly white, married, employed and had university level education. | Thematic analysis |
| Iglesias-Rosado 2021 [29] | Spain | Descriptive | Semi-structured interviews conducted online. | Breastfeeding experiences of Latina migrant women in Spain, including perceptions, challenges, and support systems | Non-probability snowball sampling of 19 women who breastfed for two months or more in the past five years recruited through online and in person support groups | Thematic analysis |
| Jackson 2021 [30] | UK | Realist | Semi-structured interviews face-to-face (in participants’ homes or research institution) and online. | Breastfeeding “beyond infancy” and how UK mothers navigate limited social and healthcare support. | Purposive sampling through local breastfeeding support group online of 24 mothers who had breastfed for more than one year. | Thematic analysis |
| Burton 2022 [31] | UK | Interpretative phenomenological analysis | Semi-structured photo-elicitation interviews and photo-prompted online surveys. | “Extended” breastfeeding and how mothers perceive their experiences. | Purposive sampling using social media recruitment of nine women who were currently breastfeeding children over 12 months. Due to the high response rate a further 15 completed the online survey. | Interpretative phenomenological analysis |
| Acquaye 2023 [32] | USA | Unspecified | Semi-structured interviews conducted online | The experiences of Black American mothers breastfeeding beyond one year | Purposive sampling recruited through Black parenting and breastfeeding support networks of 18 Black mothers who were breastfeeding for more than one year. | Content analysis |
| Augusto 2023 [33] | Portugal | Exploratory | Focus group conducted online | First-person testimonies about breastfeeding and transition to motherhood. | Convenience sampling from social media of five women aged between 31 and 44. All were highly educated and professionally active, coming from different regions of the Portuguese territory and all living with their partners of their children. | Content analysis |
| Goulden 2023 [34] | Canada | Phenomenology | Semi-structured interviews in participants’ homes and workplaces. | Infant feeding experiences of mothers who returned to work after maternity leave, with children in regulated childcare. | Convenience and snowball sampling of six participants, recruited through childcare facilities and online parenting groups, who were aged over 30, white, heterosexual, married and with some post-secondary education. | Interpretative phenomenological analysis |
| Paul 2024 [35] | Ireland | Pragmatism | Semi-structured interviews conducted online | The reported experience of women who breastfeed their children beyond one year. | Purposive sampling via social media support group for breastfeeding. A total of 14 women were recruited who had breastfed for over 1 year in the previous 2 years. The age range was 30-44 and all women were in a heterosexual relationship and were married or cohabiting with their partner. Ten lived in urban settings and four in rural settings. All were educated to third level, having attended a university or another higher education institution. Two participants were first-time mothers, and 12 had more than one child. | Thematic analysis |
| Lal-Kumar 2025 [36] | Cook Islands | Descriptive | Face-to-face qualitative interviews using the culturally grounded *Korero* method, conducted in English or Cook Islands Māori. | Feeding practices during the first 1000 days of life and the ecological factors influencing breastfeeding, weaning and infant feeding practices. | Purposive sampling of 50 parents and caregivers of children under 6 years from three Cook Islands (Rarotonga, Aitutaki and Atiu). Participants included mothers, fathers, grandparents and other caregivers recruited through flyers and word-of-mouth invitations. | Thematic analysis mapped to Bronfenbrenner's Ecological Systems Theory |
| Morgan 2025 [37] | UK | Exploratory | Semi-structured interviews conducted online via Zoom or telephone | Experiences of women breastfeeding beyond 2 years, including motivations, benefits, challenges, stigma, support and weaning experiences. | Purposive sampling through a Facebook group. Twelve mothers aged ≥18 years living in the UK who had breastfed at least one child beyond 2 years within the previous 5 years. | Thematic analysis |

**JBI Critical Appraisal Checklist for Qualitative Research**

| **Study ID (First author and year published)** | **1. Is there congruity between the stated philosophical perspective and the research methodology?** | **2. Is there congruity between the research methodology and the research question or objectives?** | **3. Is there congruity between the research methodology and the methods used to collect data?** | **4. Is there congruity between the research methodology and the representation and analysis of data?** | **5. Is there congruity between the research methodology and the interpretation of results?** | **6. Is there a statement locating the researcher culturally or theoretically?** | **7. Is the influence of the researcher on the research, and vice versa, addressed?** | **8. Are participants, and their voices, adequately represented?** | **9. Is the research ethical according to current criteria or, for recent studies, and is there evidence of ethical approval by an appropriate body?** | **10. Do the conclusions drawn in the research report flow from the analysis, or interpretation, of the data?** |
| --- | --- | --- | --- | --- | --- | --- | --- | --- | --- | --- |
| Wrigley 1990 [2] | Yes | Yes | Yes | Yes | Yes | No | No | Yes | No | Yes |
| Buckley 1992 [3] | Unclear | Yes | Yes | Yes | Yes | No | No | Yes | No | Yes |
| Marquis 1998 [4] | Yes | Yes | Yes | Yes | Unclear | No | No | Yes | Yes | Yes |
| Nelson 2005 [5] | Yes | Yes | Yes | Yes | Yes | No | No | Yes | Yes | Yes |
| Gatrell 2007 [6] | Unclear | Yes | Yes | Yes | Yes | No | No | Yes | No | Yes |
| Manhire 2007 [7] | Yes | Yes | Unclear | Yes | Yes | No | No | Yes | Yes | Yes |
| Gribble 2008 [8] | No | Yes | Yes | Yes | Yes | Yes | Unclear | Yes | Yes | Yes |
| van den Berg [9] | Unclear | Yes | Yes | Yes | Yes | No | No | Yes | No | Yes |
| Brown 2011 [10] | No | Yes | Yes | Yes | Yes | No | No | Yes | Yes | Yes |
| Demirtas 2011 [11] | Unclear | Yes | Yes | Yes | Yes | No | No | Yes | Yes | Yes |
| Stearns 2011 [12] | Unclear | Yes | Yes | Yes | Yes | Yes | Yes | Yes | Yes | Yes |
| Guyer 2012 [13] | Yes | Yes | Yes | Unclear | Yes | No | No | Yes | Yes | Yes |
| White 2012 [14] | Unclear | Yes | Yes | Yes | Yes | No | No | Yes | Yes | Yes |
| Demétrio 2013 [15] | Yes | Yes | Yes | Yes | Yes | No | No | Yes | Yes | Yes |
| Dowling 2013 [16] | Yes | Yes | Yes | Yes | Yes | Yes | Yes | Yes | Yes | Yes |
| Olanders 2013 [17] | Yes | Yes | Yes | Yes | Yes | Yes | No | Yes | Yes | Yes |
| Froh 2016 [18] | Unclear | Yes | Yes | Yes | Yes | No | No | Yes | Yes | Yes |
| Cunniff 2017 [19] | No | Yes | Yes | No | Yes | No | No | Yes | Unclear | Yes |
| Dowling 2017 [20] | Yes | Yes | Yes | Yes | Yes | Yes | Yes | Yes | Yes | Yes |
| Faircloth 2017 [21] | Yes | Yes | Yes | Yes | Yes | Yes | Yes | Yes | Unclear | Yes |
| Yate 2017 [22] | Unclear | Yes | Unclear | Yes | Yes | No | No | Yes | Unclear | Yes |
| Keevash 2018 [23] | Unclear | Yes | Yes | Yes | Yes | No | No | Yes | Yes | Yes |
| Newman 2018 [24] | Yes | Yes | Yes | Yes | Yes | No | No | Yes | Yes | Yes |
| Skelton 2018 [25] | Unclear | Yes | Yes | Yes | Yes | No | No | Yes | Yes | Yes |
| Martínez-Poblete 2020 [26] | Yes | Yes | Yes | Yes | Yes | No | No | Yes | Yes | Yes |
| Säilävaara 2020 [27] | Yes | Yes | Yes | Yes | Yes | No | No | Yes | Yes | Yes |
| Thompson 2020 [28] | Yes | Yes | Yes | Yes | Yes | Yes | Yes | Yes | Yes | Yes |
| Iglesias-Rosado 2021 [29] | Yes | Yes | Yes | Yes | Yes | No | No | Yes | Yes | Yes |
| Jackson 2021 [30] | Yes | Yes | Yes | Yes | Yes | Yes | Yes | Yes | Yes | Yes |
| Burton 2022 [31] | Yes | Yes | Yes | Yes | Yes | Yes | Yes | Yes | Yes | Yes |
| Acquaye 2023 [32] | Yes | Yes | Yes | Yes | Yes | Yes | Yes | Yes | Yes | Yes |
| Augusto 2023 [33] | Yes | Yes | Yes | Yes | Yes | No | No | Yes | Yes | Yes |
| Goulden 2023 [34] | Yes | Yes | Yes | Yes | Yes | No | Yes | Yes | Yes | Yes |
| Paul 2024 [35] | Yes | Yes | Yes | Yes | Yes | Yes | No | Yes | Yes | Yes |
| Lal-Kumar 2025 [36] | Unclear | Yes | Yes | Yes | Yes | No | No | Yes | Yes | Yes |
| Morgan 2025 [37] | Unclear | Yes | Yes | Yes | Yes | Yes | Yes | Yes | Yes | Yes |

**Full table of findings from included studies with illustrations**

| **Study ID (First author and year published)** | **Finding (Unequivocal – U, Credible – C, Not supported – NS)** | **Illustration** |
| --- | --- | --- |
| Wrigley 1990 [2] | 1. Breastfeeding on demand. (C) 2. Presencing refers to the mother being near the infant at all times. (C) 3. A relationship develops through continuous multiple interactions that take place between the long-term breastfeeding mother and her infant, as she is available to her infant through breastfeeding on demand and by presencing. (C) 4. The mothers involved in long-term breastfeeding surrender to the breastfeeding needs of the child. (C) 5. Changing priorities. (C) 6. Although the mother has reoriented her lifestyle to accommodate the relationship, she finds her ideas and beliefs in direct opposition to that of society. (C) 7. Devising a secret word. (C) 8. Limiting awareness. (U) | “*When one of them needed nursing. even If it had just been an hour. I nursed him. When one needed calming down, and needed to nurse, I let him nurse.”*  “*I tended to stay with her more and didn’t want to leave her more because of that; not because I thought she wouldn’t do well; there was just something there that I wanted to stay around her more.*”  “*I feel like I am more in touch with their needs, their emotional needs There seems to be this tie there between us so that they do not always have to verbalise what they feel. I can figure it out quicker than my friends can with their children.*”  “*I didn’t start off saying. “Well, I'm going to nurse this child a long time.” I just saw that she was a chid that had a lot of needs; she needed a lot of love and a lot of attention*."  “*I was a career woman before I had my first child, and had intended on going back to work when she was six weeks old. So breastfeeding was going to be a part-time thing for me. After [my child] was born, it just changed my life. I put my priorities in a whole different place.”*  “*I could not expect someone else to understand my feelings. I could give them reasons... but they were judging me as a person*.”  *“My daughter used to call breastfeeding “nookies.” but now she lust calls it “milkies.” People don’t know if she is asking to breastfeed or for a drink of milk.”*  “*I don’t bring up the subject with friends and family if no one else brings it up. My family now probably doesn’t even realise that she is still nursing. and I won’t say anything unless they ask.*” |
| Buckley 1992 [3] | 9. La Leche League was listed by all of the mothers as a major influence. (C) 10. The husbands were also described as an influence, but in terms of a lack of negative comments from them as opposed to the husbands directly encouraging the mothers in their practice. (C) 11. Another major influence was the child. (C) 12. Another disadvantage was the mothers' negative feelings toward the practice. (C) 13. Having to defend themselves against societal negative sanctions. (U) 14. Common responses to constraints that they felt. (NS) 15. The common sharing of sleeping arrangements between mother and child. (C) | *“[I came to be nursing a toddler] through La Leche League's work. I had gone to them with some questions when I was nursing him at about four months.”*  *“My husband, because he didn't object. He felt like I did -- that there was no reason to wean.”*  *“Number one being my child. I think once you get to being a year old, the relationship itself. It's pretty self-motivating because that is what he wants and needs. And, after a year old they make their wants and needs known pretty effectively*.”  *“I get antsy sitting there and having him just kind of -- They're not nursing to drink. They're just nursing for comfort and that can go on and on. And I get edgy. I want to go put a load of laundry in. Especially at night time when I've had no time to myself all day, I want him to finish up, get it over with so I can go do something else.”*  *“I found that when he was 15-months-old and I was nursing him in the bathroom in Macy's, and having some woman walk in with a couple of friends and notice what I was doing, and say, right in front of me, `Do you believe how big that child is, and she is nursing him?' And she just looked right at me. And I just said, `Hi.' But just like I was a thing sitting there, not like I was even present.”*  Author comments: *“The mothers employed 2 common responses to the constraints that they felt upon their behavior by their local communities and public opinion. These included the practice of some form of "closet nursing," or breastfeeding carried out with some degree of secrecy, and the mothers' encouragement of the use of a "code word" by their child for nursing.”*  *“One of the best things about extended nursing is the intimacy it gives you. It's just this closeness that we have, and even at the little bit that we do now. And sharing the bed too as a family -- that's sort of related to breastfeeding.”* |
| Marquis 1998 [4] | 16. Mothers rejected the idea that children voluntarily weaned themselves. (C) 17. Breastfeeding as a time-demanding practice. (C) 18. Recognised as an important means by which the child obtained comfort. (NS) 19. Working outside the home, and returning to a breastfeeding child were too exhausting. (NS) 20. Embarrassment about breastfeeding in public. (NS) | *“I think that in the first year they [infants] won't wean themselves, not in the second year either or in the third year. Well, my nephew was five years old and didn't wean himself. He continued breastfeeding. He continued breastfeeding. He would come back from nursery school and would look for a bench and make his mother sit down and nurse him.*”  “*They cry a lot so that all the time the mother breastfeeds them. They don't let the mother do anything.*”  Authors comments: “*Breastfeeding, however, was recognized as an important means by which the child obtained comfort, love, tranquility, security as well as communication and attention from the mother.*”  Author comments: “*Breastfeeding during the night did not allow the mother to rest completely and she would awaken tired the next day.*”  Author comments: “*Factors that were influential in the decision to wean: embarrssment about breastfeeding in public.*” |
| Nelson 2005 [5] | 21. Ending breastfeeding. (C) | Author comments: “*Although gradual weaning had advantages for Ingrid’s daughter who was able to adjust slowly to weaning at 17 months, Ingrid herself found weaning very difficult for emotional reasons. Ingrid, in spite of initially feeling relieved not to be breastfeeding any more, later said that she missed the closeness with her daughter.”* |
| Gatrell 2007 [6] | 22. Organisational antipathy towards breastfeeding, and the problems of managing leaky breasts in this situation. (U) 23. Pressure to conceal from employers their breastfeeding activities. (C) | “*I wanted to return initially for 3 days per week so I could breastfeed but permission was refused … I think they thought this would stop me breastfeeding which they said was ‘unsuitable’ at work.*”  “*I was made to feel that [breastfeeding] was unacceptable in any form … but I was determined. So I breastfed for two years because I loved breastfeeding and I know it's best for the child. So every night even though I was working (and I couldn’t express, that was out of the question), I breastfed.*” |
| Manhire 2007 [7] | 24. Conflicting beliefs, attitudes and expectations of others (families or relatives not identified) affected women’s breast feeding goals. (C) | *“My baby is now 18.5 months and still not ready to wean. In general, I have found our society works against breast feeding. I am always disappointed to hear people stop breast feeding. It is sad that not many mothers experience the delight of an 18 month old sitting up after a breast feed grinning with satisfaction”* |
| Gribble 2008 [8] | 25. Women stated that they had thought that they would only breastfeed for a short time. (C) 26. Membership of Australian Breastfeeding Association was often mentioned by women as part of the reason why they had continued breastfeeding. (U) 27. The impact of previous breastfeeding relationships. (C) | “*I was planning to feed for 6 months only as I thought that was how you did it*.”  “*At 10 months old we joined the Australian Breastfeeding Association to meet other mums who were still breastfeeding and that’s when I realized we could continue on and on…It was a combination of receiving information and support [there]… that is the reason we are still breastfeeding today.*”  “*I was more relaxed this time, had gained more knowledge and support. I learned to trust my body and its abilities.”* |
| van den Berg 2008 [9] | 28. Breastfeeding during pregnancy. (C) | *“When my son was 2 years, I was expecting my daughter. At that time, I was feeding my son. It was so difficult to stop feeding him.”* |
| Brown 2011 [10] | 29. Negative reaction of some others. (C) | *“Some friends made remarks about how they’ll still want it at 18 when I breast fed beyond a year! Quite negative, some think it weird, not right.”* |
| Demirtas 2011 [11] | 30. Influence of religion. (C) | *“If breast milk was not essential for the baby, God would not have provided it. It is useful, since it has been created by God…That’s why I wanted to breastfeed. Our religion prescribes that babies should be breastfed for two years …. I had to fulfil my obligation as a mother.”* |
| Stearns 2011 [12] | 31. Women adapting by hiding the breastfeeding. (C) 32. Breastfeeding extends beyond the boundaries of appropriate public behaviour. (C) 33. The risk of negative evaluation. (U) 34. “Code words” for nursing (C) | *“They learn who they can do it in front of and where. They have to be really discreet about how long they’re holding out because, you know they’re gonna be trashed.”*  *"People would hear him and see his actions. It made me uncomfortable for them to go, ‘Geez, he’s walking up, lifting her shirt.’"*  “*And, we were going to be going back East to visit his family a couple of weeks after she turned 2, so this was maybe a month and a half before that and I really didn’t want to have to deal with the whole stigma thing of, “You’re still nursing that baby and she’s 2 years old” kind of thing*.”  Author comments: “*The secret words, such as “milky” or “nummies,” allow the child and mother to secretly discuss breastfeeding in front of others and, if necessary, discretely negotiate delaying the breastfeeding.”* |
| Guyer 2012 [13] | 35. Mothers worried that by stopping they might upset and confuse their infant. (C) | *“Now that she’s older, I don’t know whether I’ve done the wrong thing, by now feeding her, because now she’s so aware, that it’s going to be very difficult, not just for me to stop but for her to stop it. And I think it’s going to be awful for a few nights, I really do.”* |
| White 2012 [14] | 36. Bottle feeding is for other people. (C) | *“Breastfeeding is easy, if my baby cries I can feed him straight away, bottle feeding takes a long time, bottle feeding is difficult”* |
| Demétrio 2013 [15] | 37. Breastfeeding was clearly also linked to previous positive experiences and to the family's breastfeeding experience. (C) 38. Role of breastfeeding for establishing the mother-infant bond. (C) 39. Health professional support. (C) | *“I breastfed all my children more than two years... breastfeeding is a family thing, my grandmother breastfed my mother, my mother breastfed me and my siblings, this is very important, we learn very early that we have to breastfeed our children.”*  *“I feel good when I am breastfeeding, I feel that I get closer to the child.”*  *“What I learned when I went to the hospital to have my child... what I was told is that it was important to give only breast milk until the child was 6 months old... but this child was exclusively breastfeed for 5 months ...but I continued to breastfeed her until she was 3 years old because the dietician always said that it was good to breastfeed so I continued breastfeeding.”* |
| Dowling 2013 [16] | 40. Mothers also described how those around them made the implicit assumption that once their baby had grown older they would no longer be breastfeeding. (C) 41. Mothers also described how they were often met with the reaction that what they were continuing to do was strange or abnormal behaviour. (U) 42. This reaction extended to health professionals. The majority of mothers reported that they had little support or guidance on longer-term breastfeeding, often being met by negative or crude attitudes. (C) 43. The presumption that breastfeeding was in some way a negative experience for the mother. (C) | *“About a year, when everyone else was doing something else and it was ‘‘Are you still feeding him? Are you still feeding him?’’ Now he’s eighteen months they just assume that I’ve stopped, nobody’s mentioned it for a while.”*  *“People express surprise if you are breastfeeding longer than a year, like you are a funny person.”*  *“I had a really bad earache and I’d gone back and forth to the doctor. She was prescribing something and I said ‘‘Can I take that when I’m breastfeeding?’’ and she said ‘‘How long ago did you have your baby?’’ and he was about eighteen months old, and I’m like ‘‘ages ago!’’. And they expect him to be really little. So you do kind of think, what are people thinking, you know?”*  *“‘‘Oh well, you’ve done it for so long now you don’t have to put yourself through it any more’’ and ‘‘You’ve punished yourself enough by breastfeeding her’’ and ‘‘If you don’t give up now she’s still going to be breastfeeding when she goes to school’’ and at the moment I’ve just been sort of smiling and nodding and not getting into it but then it’s a problem, isn’t it?”* |
| Olanders 2013 [17] | 44. Vulnerability. (C) 45. Outspokenness. (C) 46. Natural and normal to continue breastfeeding. (C) 47. Continuing breastfeeding made them different in the eyes of those around them. (C) | *“It almost feels like I have to defend [how we raise our son] all the time, and then I wouldn’t have the strength to defend the breastfeeding … breastfeeding feels so delicate.”*  *“I have rather felt sort of militant, that yes: I’ll show them that I’m someone who breastfeeds.”*  *"It was just obvious that he needed it. He needed the security, the closeness"*  *“I thought I was ‘normal’, I thought that I would wean her at six months… ... I began to think already when he was one year, hey, now I’m breastfeeding longer than others, how remarkable.”* |
| Froh 2016 [18] | 48. Supportive Environment/Work Culture. (C) 49. Internal Barriers. (C) | *“My nurse managers were great in allowing me adequate time to pump as often as I needed. Their support truly helped me make it to my goal of one year- and beyond.”*  *“At 22 months, there was a lot of pressure from family and friends [to stop].”* |
| Cunniff 2017 [19] | 50. The bond of breastfeeding. (NS) 51. Night feedings and cosleeping. (C) 52. Family planning and fertility. (C) 53. Support communities. (NS) | Author comments: *“All mothers noted the emotional bond that develops with their child through their breastfeeding relationship.”*  *“I don’t really know what I want at this point. I know that I don’t want to nurse her all night after 2 [years]. But I don’t really know how to do that process.”*  *“I have not gotten my period yet, which is kind of crazy...that is something, one of the reasons why I’m thinking too of [weaning night feedings], one: because we do want to have another child.”*  Author comments: “A*ll of these mothers had communities, whether online or in-person, to turn to for support and guidance.”* |
| Dowling 2017 [20] | 54. Commitment (living with long-term breastfeeding). (C) 55. Challenges in breastfeeding long-term: breastfeeding in public. (U) 56. Responding to/knowing about cultural unease with the practice. (U) 57. Breastfeeding groups were referred to as comfortable places for receiving support for breastfeeding long-term. (C) | *"…as this time continues without a clear end in sight… as the mothers of similarly aged children are returning to some state of normalcy…"*  *“…I just kind of ignore people around me, when I’m doing it… sometimes I do try and go in a bit of a quieter place… you don’t really want to be the centre of attention when you’re doing it anyway”*  *"… you’re sort of out of synch with the common view aren’t you? Cos I’m really proud that I’m still feeding her, I think it’s brilliant and it feels like totally the right thing to be doing. But it is sad, it’s sad to be in a situation where you don’t feel you can be proud of it because people don’t understand …"*  *“… there is an active home ed. group that we go to twice a month … it is like an LLL meeting because there are so many people breastfeeding both babies and toddlers! There is a lot of breastfeeding in the home ed. Community …”* |
| Faircloth 2017 [21] | 58. ‘Natural’ Parenting. (C) 59. Ecological Parenting. (NS) | *"You see, the thing is, Charlotte, what I’m doing is normal – you know, it’s actually the most natural thing in the world when you think about it. Children have a need for their mother’s milk until the age of six, and mothers are designed to fulfil that need. It feels right, because it’s part of our evolution. The benefits are in the literature."*  Authors comments: “As Ivana suggests, full-term breastfeeding is only one choice women in this sample make in cultivating an ‘ecological’ lifestyle. They often favoured the rhythm method of contraception over pills or intrauterine devices, which were understood to disrupt the body’s natural state. When they had their period, they did not use tampons or sanitary towels but a ‘menstrual cup’, which was more environmentally friendly. At birth, they would eschew ‘interventions’ and avoid painkillers where possible. Women often used cloth nappies for their children, which were said to be better for the environment (though some women countered that the detergents used to wash them were just as harmful). Children were not necessarily vaccinated, and medical treatments for illness, such as antibiotics, were used only as a last resort, as they were held to interrupt the body’s own homeostasis. The family would eat primarily organic, locally grown, whole foods if they could. They abstained from wearing synthetic or branded clothes whenever possible. They recycled. They preferred cycling to driving, and making their own food rather than buying it. As Bobel puts it (writing about the USA), ‘natural mothers’ resist culture to embrace (a cultural construction of) nature in almost all areas of their lives.” |
| Yate 2017 [22] | 60. Experiences that could be described as being agitated or irritated whilst breastfeeding. (C) 61. Maintaining breastfeeding while experiencing breastfeeding aversion and agitation. (C) | *“When my eldest son was almost 3, I wanted to wean him and I felt agitated, whenever I was feeding him.”*  *“27 months still feeding on demand, I get irritated when she is latched on.”* |
| Keevash 2018 [23] | 62. Embarrassment about the length of breastfeeding. (C) | *“I was naughty with Jordon; I breastfed Jordon until he was 26 months. He had teeth.”* |
| Newman 2018 [24] | 63. Enduring stigma from families and the community. (U) 64. ‘Weird freaky paedophiles’: media representations of extended breastfeeding. (C) 65. ‘Feeling quite exposed’: managing extended breastfeeding etiquette. (U) 66. Support from a partner or husband. (C) 67. Social media usage. (C) | *“My sister still thinks it's a bit weird that I breastfeed now because she just she never considered it, she was straight away when she found out she was pregnant it was ‘nope not doing it’ so I do get the … “She's over one now, are you really still feeding her?””*  *“For some reason, the only media coverage they show of breast-feeders are women still breastfeeding their eight year old children that are really extreme stories designed to make breast-feeders look like sort of weird freaky paedophiles… I find that really tough.”*  *“Things like you know sort of vest top under the top so your belly is not exposed at the same time… because I had to feed them kind of in the rugby ball hold round the across me a lot of the time so that was always we just knew a feeling that you were quite exposed by lifting your top up.”*  *“If he'd have just been like ‘oh just give up y'know, let's give them this, let's just give her some formula or whatever’ it would've been a lot harder for me to continue.”*  *"Lots of kind of chatting on Facebook in the middle of the night… just you know, sounding off about our night feeds and being tired and that kind of thing, so a bit of moral support there."* |
| Skelton 2018 [25] | 68. Breastfeeding in public, exclusively pumping, continued breastfeeding (past 12 months), co-sleeping, nursing at night and supplementing while breastfeeding as all having their own “taboo”. (C) | *“Women are afraid of being shamed for breastfeeding. Whether it’s for not being covered properly or the age of the child. I for one have experienced negative comments from some regarding nursing my 15 month old. Because of this, I don’t readily advertise or discuss my breastfeeding relationship outside of the groups.”* |
| Martínez-Poblete 2020 [26] | 69. Life experiences. (NS) 70. Positive feelings and emotions. (C) 71. Stimuli from the mother herself and from the child. (C) 72. Social and cultural encouragement. (C) | *“And then I told my mother I would nurse up to a year… later I thought… let’s give it a little longer and in the end I decided when she is able, until she goes to pre-school to 4 or 3 years, until she wants, I’ll nurse her...”*  *“I feel proud… and in addition I had them when I was older (laughter), then it never occurred to me to buy a can of formula… they never drank, I always had a lot of milk so I said I’m going to nurse until… as much as I can”.*  *“…breast milk is very good for them, in the sense that it complements food, meaning vitamins, minerals, calcium, hydration, it isn’t just food and hydration, but there is also the affection in between, right? ”*  *“…my husband, he watches us and he loves it. He supports me in everything, watches us and loves to see Pedro, our son, how he puts his head to nurse ...... and for the rest of the family, it’s something natural… it’s not an issue… an issue would be bottle feeding”* |
| Säilävaara 2020 [27] | 73. Breastfeeding and embodied functionality. (C) 74. Breastfeeding and embodied tiredness. (U) 75. Breastfeeding and embodied aesthetics. (C) 76. Breastfeeding and embodied pleasure. (NS) | *"I feel it is fantastic that my body can produce food for my child! My child enjoys breastfeeding and skin contact a lot, and I am happy to offer her that."*    *"The downside of breastfeeding at night is that I have not had a solid night’s sleep in two years, and little by little I am getting tired"*  *"Breastfeeding has improved my relationship with my own body. I feel more feminine. When I was younger, I was ashamed of my small breasts, but no longer—now my breasts feel precious and personal."*  Author comments: *"When pleasure was brought up, they would describe feeling close to their child, emotional pleasure, and the pleasure of doing the right thing for their child."* |
| Thompson 2020 [28] | 77. Child-led approach. (C) 78. Benefits of breastfeeding. (C) 79. Biological norm. (C) 80. Sense of achievement. (U) 81. Supporting others. (C) 82. Adjusting expectations. (C)      83. Managing perceived disapproval. (U) 84. Self-protection strategies. (U) 85. Accessing support. (NS) 86. Unable to seek professional support for fear of disapproval. (C) 87. Breastfeeding as a parenting tool. (U) | *"No one gives you a medal for breastfeeding, but I feel like I’ll get one if I can complete his breastfeeding journey on his terms…I feel like I’ll give myself one for it."*  *"When he is sick he can have my milk and I don’t have to worry quite so much about whether he’s hydrated and I know that he’ll probably get better faster."*  *"I believe in natural-term breastfeeding. It’s important that people know it’s not extended, it’s normal!"*  *"For me breastfeeding has been healing. I didn’t give birth in the way that I wanted to so being able to breastfeed has been a gift. It will be one of my greatest achievements"*  *"There's not the natural support that we would traditionally have had in the family."*  *"I think it’s very ingrained in our society that kids don’t breastfeed: Babies wean onto solids and that’s the end of it. That’s what I thought happened. I didn’t realise it [lactation] carried on."*  *"I feel very under the microscope since I’ve come back to work. I’ve had comments like ‘well you’re still feeding her, what do you expect? She’s still using you as a dummy"*  *"When I picked her up from nursery she would always want a feed and I didn’t just feed her there, I would go and hide somewhere…I didn’t even tell the nursery staff I was still breastfeeding."*  Author comments: *"Many women attended in-person support groups, but online groups became increasingly important as children aged."*  *"The consultant made some comments about how I should be considering weaning and not feeding my baby anymore because of her age… he told me there were no benefits to breastfeeding beyond two, and he told me that breastfeeding hinders children’s development."*  *"If he does get really upset about something and he can’t calm down usually he can settle with having a little bit of milk. So for me it’s like this cure all—it’s so wonderful; I rely on it quite a lot."* |
| Iglesias-Rosado 2021 [29] | 88. Support networks: partner, health professionals, and family. (C) | *“If he [husband] hadn’t made this big effort with me, I probably wouldn’t have been nursing for 25 months.”* |
| Jackson 2021 [30] | 89. As their breastfeeding continued, support dwindled. (C) 90. Women started to experience judgemental glances from strangers when feeding in public and their breastfeeding practices were challenged more directly by friends and family. (C) 91. General Practitioners (GP) and other healthcare professionals also became less supportive. (U) 92. La Leche League meetings offered women the space to meet up. (U) 93. Groups set up on social media specifically for women breastfeeding beyond infancy. (U) | *"No one can object to new-born breastfeeding because that is their only food source but I think people feel differently when they can eat or drink other things."*  "*She’s like, “You don’t want him to be four and asking for milk,” and it’s just like, “Why not?” It’s like the older they get you become like a closet breastfeeder.*”  *"I suffered with vertigo at the beginning of the year and this doctor said to take these tablets you need to stop breastfeeding and it’s about time you need to stop breastfeeding anyway but I didn’t."*  *"It’s completely normal, completely natural, and I think having seen people feeding children that were eighteen months, two years, it made me realise it’s not weird, its completely normal. It had a major influence."*  *“There’s a really good group I’m part of, Breastfeeding Babies and Beyond, I think it is, and they’re from all over the world, and there’s thousands of us and you can post anything and you’ll get a response pretty much straight away.”* |
| Burton 2022 [31] | 94. It's about closeness and connection. (U) 95. Parenting through breastfeeding: meeting the needs of my child. (U) 96. My body is not my own. (U) 97. Social support and role modelling. (U) 98. Perceived societal views. (U) 99. Thinking about stopping: my choice or theirs. (U) | *"It’s just that connection, it’s just that connection, that quiet time for the two of you, neither of you are kind of distracted with anything else. It’s just a nice kind of snuggle."*  *"Boobs solve everything. Tired? Boobs. Cranky? Boobs. Hyper? Boobs. Sad? Angry? Scared? Hungry?.... it’s always boobs […]It’s an easy way to parent. I don’t know how to be a parent without boobs."*  *"[This photograph] represents the part which makes me sometimes think about stopping feeding, he gets very active, climbs over me will lie on my throat and generally it becomes an uncomfortable experience for both myself and my husband as he starts to kick out sideways."*  *"I think it’s really nice to, to know different mums at different stages, because it, it shows how it can fit into your life, because at the very beginning you think, you’re going to be attached to this baby for the next like another two years and you are never going to be able to leave the house or wash your hair without, you know, but when you actually speak to mums at different stages and you realise that that is not the case."*  *"[I] chose that [photograph] because it was one out and about, which I feel as your child gets older breastfeeding out and about is a little bit more kind of frowned upon. I have never ever had any comments, but I do feel a little bit more conscious now that she is older of what people might be thinking. […] I don’t see other people feeding a child that age very often, and I think people expect you to have stopped by the time your child is that age."*  *"With my first daughter I really found breastfeeding harder towards the end, wanted my body back and in the end we ended the feeding probably a bit to abruptly, (at age 2 years and 1 month), and I do regret that in some ways. This time I’ve decided to go on for a bit longer, although at some point I would like to start gently weaning. I would love for things to come to a natural end but I am not sure I can feed for another 18 months if that’s how long it will take."* |
| Acquaye 2023 [32] | 100. Deciding to continue beyond 12 months. (C) 101. Pressure to stop breastfeeding. (U) 102. Support to continue breastfeeding. (C) 103. Overall struggles. (C) | *"To be continuing to breastfeed now at 18 months, I think it’s just at that point where it’s just like, we’re just going to go until he says he doesn’t want it anymore or until it’s just too much. But I think every day, every couple of days, I kind of start to rethink it like am I ready to wean and my—because he’s not ready to wean, but am I?’’*  *"[The pediatrician] made a comment that really [sighs] she said she asked me, ‘so how much milk is [your] daughter drinking?’ And I think she was three at the time. So I said, ‘I don’t know, she just drinks the breast milk. And she’s like, she said, ‘Oh, so you give her from the boob.’ I said, ‘yes’. And then she said, ‘we usually wean at one.’’’*  *"When I think about a support system now when it comes to breastfeeding, I will wholeheartedly have to say the number one support system that I came into contact with that was uh lifesaving was the online platform for Black moms. That was huge."*  *"Me and my aunt got into it, we stopped talking for almost a year because she was watching [my son] and we told her do not give my baby cereal. [My aunt said] ‘Ah the baby needs it’. She kept pressing and pressing, I’m like, ‘no’. She snuck and gave that baby cereal. You know how [I knew]? Because when you breastfeed a baby their poop, you know their poop you know it’s smell. I looked in that diaper and it had like little remnants. I took a picture of it. It had a different smell. I said she gave that baby—!’’* |
| Augusto 2023 [33] | 104. The decision to stop breastfeeding seems to be subject to much more reflection, as it is viewed with some reluctance and as a source of anxiety and suffering. (NS) 105. Pressure to weaning, to stop breastfeeding. (C) 106. Lack of traditional support for their mothering practices—family, friends, close community, neighbourhood—as an explanation to seek specialised support. (C) 107. Experiences of conciliation between the return to work and breastfeeding were diverse. (U)      108. Intimacy and couple life. (C) 109. The role of the father in breastfeeding. (C) 110. Changes in self-concept and processes of identity. (C) | Authors comments: *“Of all the participants, only two had stopped breastfeeding at the moment of the interview, one of them not by choice.”*  *“I often hear ‘But is he still breastfeeding? He's too old to still breastfeed.’ And I'm not talking about outsiders. The opinions of those closest to me are the most difficult ones, and even when my husband says that this has to stop, because he doesn't sleep. In those moments I really feel a stab in the back. (...) I feel this pressure from my husband, but the times I wake up, he's sleeping. And even if the nights are bad, I have the right to continue not sleeping and breastfeeding. But it's getting harder and harder because I feel like I have no way to argue. How do I say that this thing I want to do is something I want to do?”*  *“I found it very strange not to share this moment in life. What an enormous silence there is among women! I don't have siblings; I don't have close cousins either. In my family I did not live other experiences of motherhood, in my friendships neither (...). I was a little disappointed [with the lack of a close support network]”*  *“I felt pressure from my employer because I pumped milk during my breaks, then I came home to lunch and breastfed my baby. Then, in the afternoon, I pumped again during the break. (...) It seemed like I was always expressing milk [in the workplace], but I only did it during my breaks and sometimes I didn't even stop working. But I received some comments that, in my opinion, were a little unnecessary. (..) When my son was already 16 months old, I realized that I was under too much pressure at work and (...), since I had some milk in stock, I decided to stop pumping milk at work.”*  *“Like Alice, I also feel that the breast, which used to be, so to speak, an object of pleasure, has become completely dominated by the child. (...) For me it was very difficult, because the breast for me has always been a very erogenous zone and after childbirth that disappeared. It cost me a lot because for me it was always a part of my body that gave me a lot of pleasure. It was as if breastfeeding had stolen that from me.”*  *“I felt that in the beginning there was much more participation and support [from my husband]. (...) Then, with exhaustion and the passage of time, he stopped waking up at night or no longer got up every time I did. (...) It's difficult because when I put the baby to the breast, he falls asleep, but if the father has to cradle him, it takes forever to fall asleep, so I also had responsibility because I didn't promote his participation. It is something we will certainly do differently next time!”*  *“I have always had a complex relationship with my breasts. [Breastfeeding and mothering] helped me to overcome some things in my relationship with my body that, despite my age, were still there. This whole process, which is extremely physical, contributed to the acceptance, to understand how the body itself can be challenged. And after 3 years, despite this speech, I feel that I am only now getting my body back”* |
| Goulden 2023 [34] | 111. Weaning stress. (C) | *“After he had been in the child care center for about a month, when he was 13 months old, I was confused and worried that he should be drinking more [breast] milk since I’m not around as much … So, we did at 13 months give him cow’s milk and that took some of the stress away too.”* |
| Paul 2024 [35] | 112. Familial influences on the women’s breastfeeding decisions and practices were reported to be very important. Friends were also identified as key influencers. (C) 113. The women perceived society’s view on breastfeeding a toddler as negative. (U) 114. Reluctant to breastfeed their toddler in public (C) 115. Women identified the marketing of commercial milk formula as negatively influencing breastfeeding beyond 1 year. (C) 116. Partners provided emotional support by encouraging the women to continue and providing reassurance through challenging periods. (U) 117. The women accessed a range of breastfeeding support groups, including La Leche League and Public Health Nursing (PHN)- led groups which they found very supportive. (U) 118. Workplace supports for breastfeeding beyond 1 year varied. (U) 119. Women experienced a lack of support and understanding from health professionals when interacting with the health service as mothers who were breastfeeding a toddler. (U) 120. There was a sense of achievement in continuing to breastfeed beyond their child’s first birthday. (U) 121. Knowledge improved as their breastfeeding journey progressed. (U) 122. All the women reported sharing a bed with their toddler, mainly to facilitate breastfeeding but also to reduce the disruption to their sleep. (U) 123. Breastfeeding beyond 1 year was seen among other things as a great support to parenting. (C) | *"Maybe it’s the bubble that I live in, I don’t know I hope it’s not, but I have experienced quite a few friends that have breastfed long term."*  *"I just think that culturally there’s such a negative association with it [breastfeeding beyond one year] that it does massively impact on the amount of people who do it for longer than six months, so it’s just total lack of education, like, for me, I didn’t even know you did feed after six months."*  *“Like, he is now a big tall child. While I would definitely still feed him if he was upset or if he asked for it I would but like I would never offer it to him out. Because I would just feel like, I think I would feel that people are watching me.”*  *"I would have seen an awful lot of pop ups coming up on my social media for [two names of CMF] and things like that, until I physically went and changed my settings so that I wouldn’t see them anymore"*  *"He was very supportive and keep pushing me when I felt like I can’t do it anymore, or whatever like, he was ‘you can, you can, you can just one day at a time.*”  *"It is an amazing resource- just knowing people are doing the same thing. If you are feeding children later on you are more likely that something is going to come up that you need to check it is ok."*  *"Everything comes down to your individual manager. It doesn’t really matter about the company or their policies or anything it comes down to individual managers being supportive or not."*  *"I wasn’t even given anything to express into you know that’s why I ended up being in such a desperate position that I was expressing into the sink. I was in agony, but I found like there was no sympathy, no support, no acknowledgement to this whatsoever- a bit from the nurses, but not from the doctors."*  *"Oh, I’m definitely aiming for the two years now…if I could hit the two years I’d be quite proud of myself. I am very determined when I want to be."*  *"I knew all that information about how good it is for the babies before I started breastfeeding, but it’s only as I’ve become interested in it that I’ve realized in terms of kind of that it reduces your own chance of breast cancer and things like that I never quite realized that early on."*  *"I co sleep and I still do co sleep with the twins. I think, like, most people who breastfeed twins will co sleep, it’s so hard to breastfeed otherwise I don’t know how you manage."*  *"I just think it provides a soothing balm if there’s any kind of upset or anything in a way that not many other things can do."* |
| Lal-Kumar 2025 [36] | 124. Child-led weaning. (C) | *“I am still breastfeeding our youngest, he is 2 years old now, I will stop when he doesn't want it anymore like all my older kids.”* |
| Morgan 2025 [37] | 125. Breastfeeding benefited their lifestyle and complimented their parenting philosophy. (U) 126. Breastfeeding was also used as a parenting tool. (U) 127. Opportunity for bonding and reconnecting. (U) 128. Physical benefits for their child. (U) 129. Benefits of breastfeeding to the mother's own physical and mental health. (U) 130. Social media was used as a source of information. (U) 131. Solidarity with other women. (U) 132. Desire to ‘normalise’ breastfeeding older children. (U) 133. Judgement from their family and pressure to stop breastfeeding (U) 134. Influence a woman's partner had on her decision to continue feeding. (U) 135. Society's support for breastfeeding around the age of one. (U) 136. Judgemental or ill‐informed comments coming from healthcare professionals. (U) 137. Avoiding breastfeeding in public due to the social stigma. (U) 138. Lack of bodily autonomy. (U) 139. Implementing boundaries. (U) 140. How difficult it was to stop breastfeeding before their child was ready. (U) 141. Aversion towards feeding their older child. (U) | *“It's an easy way for me to parent. Yeah, it's just the easier way for me … if I said no to him it would mean a lot more meltdowns. And you know, we gentle parent as much as we can.”*  *“Literally it just it worked for everything. Like if he was upset or crying … Boob. If he was hurt. Boob. Why would I stop doing something that you know, just calms him down in any situation?”*  *“When I went back to work it was always really nice to be able to like have that as our way of being like “Right we're back together now.” This is our thing.”*  *“The milk changes … I didn't know any of this but the hormones in it from morning to night are different to help drill in the rhythms to your baby … Lipid content as well, things like where their brain has massive leaps it puts way more whey into the milk that's supporting brain development, as opposed to casein that are more for muscle development.”*  *“It's great for me, you know? Because it's going to help me reduce the chances of X, Y and Z in the future … Like cancer and obesity and all these things.”*  *“I'm on the global breastfeeding beyond page, which I find very helpful. And I'm so glad it exists because I think it normalises something that women probably feel like, like perhaps they're on their own. There's the most amazing posts that people share, like research and things.”*  *“Anybody else who'd kind of breastfed for like a longer amount of time would be like, “Oh my God, I breastfed mine until they were four.” And, you know, like there was this instant bond.”*  *“I feel like I've got a duty in a way, to make it visible and help educate people for the future … like to help women coming through behind me.”*  *“My family are like “Oh you're still feeding him” and I remind them again about the advice and guidance and say “You can look it on the Internet. A lot of things have changed since you know, we were kids.””*  *“Like he didn't actually know I was still feeding my son when my daughter arrived because he was like you need to stop now. So I just went “Yeah, OK … no worries.” And I just … I'm always up before him, so I just used to still feed him in the morning. I think I carried on feeding him for another four months after that.”*  *“When he was nearing one, I started getting a lot more questions. “When are you gonna stop feeding him? Why are you still feeding him?” Questions like that.”*  *“Yeah, my health visitor told me how I'd still be feeding him at the school gate and how he'd be asking me for bitty in reference to, you know, Little Britain.”*  *“I do tend to avoid it if I can or distract him. Honestly … it is the stigma around it. And also, he's older so I can tell him “Look, we'll have some when we get home” or I make him wait until we're back at the car.”*  *“I think it's really taxing on your body. Like, it's all consuming. Even when you're not doing it very often, it's still like a really bodily experience. And then when we finally stopped I was relieved because I had wanted that kind of … like my body back.”*  *“As he's got a bit older now, I've definitely had to set more boundaries with nursing. So it will be “We'll play one song and you can nurse for the song, and then you've got to come off.” I had to kind of develop tactics to try and get him off.”*  *“People don't understand it until they've done it. And everyone's like “Well, it's easy to just stop, isn't it?” And it's … it's really not just easy to stop breastfeeding. There's a big misconception about just stopping one day, and that's it.”*  *“It feels like my skin is crawling and like … I just feel like I literally wanna push his face off me. It's the most horrible feeling ever. You know, unless you've had aversion, you just honestly can't understand how horrible the feeling is.”* |

**Findings and associated categories**

| **Findings** | **Categories** |
| --- | --- |
| 6. Although the mother has reoriented her lifestyle to accommodate the relationship, she finds her ideas and beliefs in direct opposition to that of society. (C)  13. Having to defend themselves against societal negative sanctions. (U)  24. Conflicting beliefs, attitudes and expectations of others (families or relatives not identified) affected women’s breast feeding goals. (C)  32. Breastfeeding extends beyond the boundaries of appropriate public behaviour. (C)  33. The risk of negative evaluation. (U)  29. Negative reaction of some others. (C)  30. Influence of religion. (C)  40. Mothers also described how those around them made the implicit assumption that once their baby had grown older they would no longer be breastfeeding. (C)  41. Mothers also described how they were often met with the reaction that what they were continuing to do was strange or abnormal behaviour. (U)  43. The presumption that breastfeeding was in some way a negative experience for the mother. (C)  47. Continuing breastfeeding made them different in the eyes of those around them. (C)  56. Responding to/knowing about cultural unease with the practice. (U)  63. Enduring stigma from families and the community. (U)  64. ‘Weird freaky paedophiles’: media representations of extended breastfeeding. (C)  83. Managing perceived disapproval. (U)  62. Embarrassment about the length of breastfeeding. (C)  89. As their breastfeeding continued, support dwindled. (C)  90. Women started to experience judgemental glances from strangers when feeding in public and their breastfeeding practices were challenged more directly by friends and family. (C)  68. Breastfeeding in public, exclusively pumping, continued breastfeeding (past 12 months), co-sleeping, nursing at night and supplementing while breastfeeding as all having their own “taboo”. (C)  72. Social and cultural encouragement. (C)  98. Perceived societal views. (U)  76. Pressure to stop breastfeeding. (U)  105. Pressure to weaning, to stop breastfeeding. (C)  113. The women perceived society’s view on breastfeeding a toddler as negative. (U)  114. Reluctant to breastfeed their toddler in public (C)  135. Society's support for breastfeeding around the age of one. (U)  137. Avoiding breastfeeding in public due to the social stigma. (U) | Social judgement, stigma and cultural perceptions |
| 7. Devising a secret word. (C)  8. Limiting awareness. (U)  31. Women adapting by hiding the breastfeeding. (C)  34. “Code words” for nursing (C)  44. Vulnerability. (C)  47. Continuing breastfeeding made them different in the eyes of those around them. (C)  55. Challenges in breastfeeding long-term: breastfeeding in public. (U)  65. ‘Feeling quite exposed’: managing extended breastfeeding etiquette. (U)  84. Self-protection strategies. (U)  114. Reluctant to breastfeed their toddler in public (C)  115. Women identified the marketing of commercial milk formula as negatively influencing breastfeeding beyond 1 year. (C)  132. Desire to ‘normalise’ breastfeeding older children. (U | Public visibility and self-protection strategies |
| 22. Organisational antipathy towards breastfeeding, and the problems of managing leaky breasts in this situation. (U)  23. Pressure to conceal from employers their breastfeeding activities. (C)  48. Supportive Environment/Work Culture. (C)  107. Experiences of conciliation between the return to work and breastfeeding were diverse. (U)  118. Workplace supports for breastfeeding beyond 1 year varied. (U) | Influence of the workplace |
| 42. This reaction extended to health professionals. The majority of mothers reported that they had little support or guidance on longer-term breastfeeding, often being met by negative or crude attitudes. (C)  39. Health professional support. (C)  86. Unable to seek professional support for fear of disapproval. (C)  91. General Practitioners (GP) and other healthcare professionals also became less supportive. (U)  76. Pressure to stop breastfeeding. (U)  117. The women accessed a range of breastfeeding support groups, including La Leche League and Public Health Nursing (PHN)- led groups which they found very supportive. (U)  119. Women experienced a lack of support and understanding from health professionals when interacting with the health service as mothers who were breastfeeding a toddler. (U)  136. Judgemental or ill‐informed comments coming from healthcare professionals. (U) | Healthcare professional support |
| 9. La Leche League was listed by all of the mothers as a major influence. (C)  26. Membership of Australian Breastfeeding Association was often mentioned by women as part of the reason why they had continued breastfeeding. (U)  57. Breastfeeding groups were referred to as comfortable places for receiving support for breastfeeding long-term. (C)  67. Social media usage. (C)  81. Supporting others. (C)  92. La Leche League meetings offered women the space to meet up. (U)  93. Groups set up on social media specifically for women breastfeeding beyond infancy. (U)  97. Social support and role modelling. (U)  102. Support to continue breastfeeding. (C)  106. Lack of traditional support for their mothering practices—family, friends, close community, neighbourhood—as an explanation to seek specialised support. (C)  117. The women accessed a range of breastfeeding support groups, including La Leche League and Public Health Nursing (PHN)- led groups which they found very supportive. (U)  130. Social media was used as a source of information. (U) | Role of breastfeeding organisations and peer support |
| 10. The husbands were also described as an influence, but in terms of a lack of negative comments from them as opposed to the husbands directly encouraging the mothers in their practice. (C)  33. The risk of negative evaluation. (U)  37. Breastfeeding was clearly also linked to previous positive experiences and to the family's breastfeeding experience. (C)  63. Enduring stigma from families and the community. (U)  66. Support from a partner or husband. (C)  49. Internal Barriers. (C)  90. Women started to experience judgemental glances from strangers when feeding in public and their breastfeeding practices were challenged more directly by friends and family. (C)  72. Social and cultural encouragement. (C)  103. Overall struggles. (C)  105. Pressure to weaning, to stop breastfeeding. (C)  106. Lack of traditional support for their mothering practices—family, friends, close community, neighbourhood—as an explanation to seek specialised support. (C)  109. The role of the father in breastfeeding. (C)  112. Familial influences on the women’s breastfeeding decisions and practices were reported to be very important. Friends were also identified as key influencers. (C)  88. Support networks: partner, health professionals, and family. (C)  116. Partners provided emotional support by encouraging the women to continue and providing reassurance through challenging periods. (U)  133. Judgement from their family and pressure to stop breastfeeding (U)  134. Influence a woman's partner had on her decision to continue feeding. (U) | Influence of partners, family and friends |
| 1. Breastfeeding on demand. (C)  2. Presencing refers to the mother being near the infant at all times. (C)  3. A relationship develops through continuous multiple interactions that take place between the long-term breastfeeding mother and her infant, as she is available to her infant through breastfeeding on demand and by presencing. (C)  11. Another major influence was the child. (C)  36. Bottle feeding is for other people. (C)  38. Role of breastfeeding for establishing the mother-infant bond. (C)  78. Benefits of breastfeeding. (C)  87. Breastfeeding as a parenting tool. (U)  94. It's about closeness and connection. (U)  70. Positive feelings and emotions. (C)  71. Stimuli from the mother herself and from the child. (C)  95. Parenting through breastfeeding: meeting the needs of my child. (U)  123. Breastfeeding beyond 1 year was seen among other things as a great support to parenting. (C)  126. Breastfeeding was also used as a parenting tool. (U)  127. Opportunity for bonding and reconnecting. (U) | Breastfeeding as a parenting tool and emotional bond |
| 5. Changing priorities. (C)  45. Outspokenness. (C)  46. Natural and normal to continue breastfeeding. (C)  58. ‘Natural’ Parenting. (C)  73. Breastfeeding and embodied functionality. (C)  75. Breastfeeding and embodied aesthetics. (C)  79. Biological norm. (C)  80. Sense of achievement. (U)  108. Intimacy and couple life. (C)  110. Changes in self-concept and processes of identity. (C)  120. There was a sense of achievement in continuing to breastfeed beyond their child’s first birthday. (U)  125. Breastfeeding benefited their lifestyle and complimented their parenting philosophy. (U)  131. Solidarity with other women. (U) | Identity and empowerment |
| 2. Presencing refers to the mother being near the infant at all times. (C)  4. The mothers involved in long-term breastfeeding surrender to the breastfeeding needs of the child. (C)  12. Another disadvantage was the mothers' negative feelings toward the practice. (C)  17. Breastfeeding as a time-demanding practice. (C)  28. Breastfeeding during pregnancy. (C)  74. Breastfeeding and embodied tiredness. (U)  51. Night feedings and cosleeping. (C)  52. Family planning and fertility. (C)  60. Experiences that could be described as being agitated or irritated whilst breastfeeding. (C)  61. Maintaining breastfeeding while experiencing breastfeeding aversion and agitation. (C)  96. My body is not my own. (U)  108. Intimacy and couple life. (C)  122. All the women reported sharing a bed with their toddler, mainly to facilitate breastfeeding but also to reduce the disruption to their sleep. (U)  138. Lack of bodily autonomy. (U)  141. Aversion towards feeding their older child. (U) | Physical and emotional challenges |
| 4. The mothers involved in long-term breastfeeding surrender to the breastfeeding needs of the child. (C)  15. The common sharing of sleeping arrangements between mother and child. (C)  16. Mothers rejected the idea that children voluntarily weaned themselves. (C)  21. Ending breastfeeding (C)  25. Women stated that they had thought that they would only breastfeed for a short time. (C)  27. The impact of previous breastfeeding relationships. (C)  35. Mothers worried that by stopping they might upset and confuse their infant. (C)  46. Natural and normal to continue breastfeeding. (C)  54. Commitment (living with long-term breastfeeding). (C)  58. ‘Natural’ Parenting. (C)  77. Child-led approach. (C)  79. Biological norm. (C)  82. Adjusting expectations. (C)  99. Thinking about stopping: my choice or theirs. (U)  100. Deciding to continue beyond 12 months. (C)  121. Knowledge improved as their breastfeeding journey progressed. (U)  122. All the women reported sharing a bed with their toddler, mainly to facilitate breastfeeding but also to reduce the disruption to their sleep. (U)  111. Weaning stress. (C)  124. Child-led weaning. (C)  128. Physical benefits for their child. (U)  129. Benefits of breastfeeding to the mother's own physical and mental health. (U)  139. Implementing boundaries. (U)  140. How difficult it was to stop breastfeeding before their child was ready. (U) | Motivation and adaptation |

**References**

1.	Malterud K. Qualitative research: standards, challenges, and guidelines. Lancet 2001;358:483–488

2.	Wrigley EA, Hutchinson SA. Long-term breastfeeding. The secret bond. J Nurse Midwifery 1990;35:35–41

3.	Buckley KM. Beliefs and practices related to extended breastfeeding among La Leche League mothers. Journal of perinatal education. 1992 Jun 1;1(2).

4.	Marquis GS, Díaz J, Bartolini R, Creed De Kanashiro H, Rasmussen KM. Recognizing the reversible nature of child-feeding decisions: Breastfeeding, weaning, and relactation patterns in a shanty town community of Lima, Peru. Soc Sci Med 1998;47:645–656

5.	Nelson A, Sethi S. The breastfeeding experiences of Canadian teenage mothers. JOGNN - J Obstet Gynecol Neonatal Nurs 2005;34:615–624

6.	Gatrell CJ. Secrets and lies: Breastfeeding and professional paid work. Soc Sci Med 2007;65:393–404

7.	Manhire KM, Hagan AE, Floyd SA. A descriptive account of New Zealand mothers’ responses to open-ended questions on their breast feeding experiences. Midwifery 2007;23:372–381

8.	Gribble KD. Long-term breastfeeding; changing attitudes and overcoming challenges. Breastfeed Rev 2008;16:5–15

9.	van den Berg M, Ball HL. Practices, advice and support regarding prolonged breastfeeding: A descriptive study from Sri Lanka. J Reprod Infant Psychol 2008;26:229–243

10.	Brown A, Raynor P, Lee M. Young mothers who choose to breast feed: The importance of being part of a supportive breast-feeding community. Midwifery 2011;27:53–59

11.	Demirtas B, Ergocmen B, Taskin L. Breastfeeding experiences of Turkish women. J Clin Nurs 2012;21:1109–1118

12.	Stearns CA. Cautionary tales about extended breastfeeding and weaning. Health Care Women Int 2011;32:538–554

13.	Guyer J, Millward LJ, Berger I. Mothers’ breastfeeding experiences and implications for professionals. Br J Midwifery 2012;20:724–733

14.	White AL, Carrara VI, Paw MK, et al. High initiation and long duration of breastfeeding despite absence of early skin-to-skin contact in Karen refugees on the Thai-Myanmar border: a mixed methods study. Int Breastfeed J 2012;7:19

15.	Demétrio F, Silva M da CM da, Chaves-dos-Santos SM, Assis AMO. Meanings attributed to breastfeeding in the first two years of life: a study with women from two municipalities in the Recôncavo Baiano region of Bahia, Brazil. Rev Nutr 2013;26:5–16

16.	Dowling S, Brown A. An exploration of the experiences of mothers who breastfeed long-term: What are the issues and why does it matter? Breastfeed Med 2013;8:45–52

17.	Olanders M. Breastfeeding feels so delicate How mothers of breastfed preschool children experience being treated by preschool teachers. Breastfeed. Rev. . 2013. M. Olanders, 21: 45–52

18.	Froh EB, Spatz DL. Navigating return to work and breastfeeding in a hospital with a comprehensive employee lactation program: the voices of mothers. J Hum Lact 2016;32:689–694

19.	Cunniff A, Spatz D. Mothers’ Weaning Practices when Infants Breastfeed for More Than One Year. MCN Am J Matern Child Nurs 2017;42:88–94

20.	Dowling S, Pontin D. Using liminality to understand mothers’ experiences of long-term breastfeeding: “Betwixt and between”, and “matter out of place.” Health (London) 2017;21:57–75

21.	Faircloth, C. 2017. “‘Natural’ Breastfeeding in Comparative Perspective: Feminism, Morality, and Adaptive Accountability.” Ethnos: Journal of Anthropology 82 (1, February): 19. doi:10.1080/00141844.2015.1028562.

22.	Yate ZM. A qualitative study on negative emotions triggered by breastfeeding; describing the phenomenon of breastfeeding/nursing aversion and agitation in breastfeeding mothers. Iran J Nurs Midwifery Res 2017;22:449

23.	Keevash J, Norman A, Forrest H, Mortimer S. What influences women to stop or continue breastfeeding? A thematic analysis. Br J Midwifery 2018;26:651–658

24.	Newman KL, Williamson IR. Why aren’t you stopping now?!’ Exploring accounts of white women breastfeeding beyond six months in the East of England. Appetite 2018;129:228–235

25.	Skelton KR, Evans R, LaChenaye J, Amsbary J, Wingate M, Talbott L. Exploring social media group use among breastfeeding mothers: Qualitative analysis. JMIR Pediatr Parent 2018;1:e11344

26.	Martínez-Poblete G, Ossa X. Motivations for breastfeeding prolongation. Acta Paul Enferm 2020;33:eAPE20190112

27.	Säilävaara J. Long-term breastfeeding: the embodied experiences of Finnish mothers. NORA-Nordic J Fem Gend Res 2020;28:43–55

28.	Thompson AJ, Topping AE, Jones LL. Surely you’re not still breastfeeding’: A qualitative exploration of women’s experiences of breastfeeding beyond infancy in the UK. BMJ Open 2020;10

29.	Iglesias-Rosado B, Leon-Larios F. Breastfeeding experiences of Latina migrants living in Spain: a qualitative descriptive study. Int Breastfeed J 2021;16:76

30.	Jackson JE, Hallam J. Against all odds-why UK mothers’ breastfeeding beyond infancy are turning to their international peers for emotional and informative support. Health Care Women Int 2021;42:739–755

31.	Burton AE, Taylor J, Owen AL, Renshaw JE, Williams LR, Dean SE. A photo-elicitation exploration of UK mothers’ experiences of extended breastfeeding. Appetite 2022;169

32.	Acquaye SN, Spatz DL. Lactation Experiences of Black Mothers Who Breastfed a Child Beyond Age One. Breastfeed Med 2023;18:326–333

33.	Augusto A, Neves DM, Henriques V. Breastfeeding experiences and women’s self-concept: Negotiations and dilemmas in the transition to motherhood. Front Sociol 2023;8:1130808

34.	Goulden A, Mann L, Norris D, Rossiter M. Working mothers’ infant feeding experiences during their children’s transition to child care: a qualitative study. J Child Fam Stud 2023;32:2214–2228

35.	Paul G, Vickers N, Kincaid R, McGuinness D. ‘It’s far from the norm’: breastfeeding beyond 1 year in the Republic of Ireland. Health Promot Int 2024;39:daae088

36.	Lal‐Kumar S, Dayal H, Seve‐Williams N, Morse Z, Zafar S. The First 1000 Days of Life: Breastfeeding in The Cook Islands—An Ecological Perspective Using Bronfenbrenner’s Model. International Journal of Paediatric Dentistry. 2026 Jan;36(1):67-73.

37.	Morgan J, Jones S, Brown A. Exploring the experiences and challenges of breastfeeding beyond 2 years in the United Kingdom: A qualitative study. Maternal & Child Nutrition. 2026 Jan;22(1):e70072.
